# Synthesis, Structure, Chemical Stability, and In Vitro Cytotoxic Properties of Novel Quinoline-3-Carbaldehyde Hydrazones Bearing a 1,2,4-Triazole or Benzotriazole Moiety

**DOI:** 10.3390/molecules23061497

**Published:** 2018-06-20

**Authors:** Martyna Korcz, Franciszek Sączewski, Patrick J. Bednarski, Anita Kornicka

**Affiliations:** 1 Department of Chemical Technology of Drugs, Medical University of Gdańsk, Al. Gen. J. Hallera 107, 80-416 Gdańsk, Poland; martyna.korcz@wp.pl (M.K.); a.kornicka@gumed.edu.pl (A.K.); 2 Department of Pharmaceutical and Medicinal Chemistry, Institute of Pharmacy, University of Greifswald, F.-L. Jahn Strasse 17, D-17489 Greifswald, Germany; bednarsk@uni-greifswald.de

**Keywords:** quinolines, 1,2,4-triazoles, 1,2,3-benzotriazoles, hydrazones, *N*-acylhydrazones, *N*-sulfonylhydrazones, synthesis, structure, in vitro antitumor activity

## Abstract

A small library of novel quinoline-3-carbaldehyde hydrazones (Series 1), acylhydrazones (Series 2), and arylsulfonylhydrazones (Series 3) bearing either a 1,2,4-triazole or benzotriazole ring at position 2 was prepared, characterized by elemental analyses and IR, NMR, and MS spectra, and then subjected to in vitro cytotoxicity studies on three human tumor cell lines: DAN-G, LCLC-103H, and SISO. In general, compounds **4**, **6**, and **8** substituted with a 1,2,4-triazole ring proved to be inactive, whereas the benzotriazole-containing quinolines **5**, **7**, and **9** elicited pronounced cancer cell growth inhibitory effects with IC_50_ values in the range of 1.23–7.39 µM. The most potent 2-(1*H*-benzotriazol-1-yl)-3-[2-(pyridin-2-yl)hydrazonomethyl]quinoline (**5e**) showed a cytostatic effect on the cancer cell lines, whereas *N*′-[(2-(1*H*-benzotriazol-1-yl)quinolin-3-yl)methylene]-benzohydrazide (**7a**) and *N*′-[(2-1*H*-benzotriazol-1-yl)quinolin-3-yl)methylene]-naphthalene-2-sulfonohydrazide (**9h**) exhibited selective activity against the pancreas cancer DAN-G and cervical cancer SISO cell lines. Based on the determined IC_50_ values, the compound **5e** seems to be leading compound for further development as anticancer agent.

## 1. Introduction

The term “privileged structures” was coined by Evans and co-workers [1] and since then has proven to be an effective approach in drug discovery process [2,3]. Among the reported privileged structures, the quinoline scaffold constitutes one of the most explored heterocyclic systems due to its broad range of pharmacological activities [4,5,6,7]. Of special interest are the anticancer properties of quinoline derivatives [8,9,10]. Thus, the quinoline ring is utilized in clinically used anticancer drugs, such as *camptothecin* and its analogues, e.g., *topotecan*, which are known as topoisomerase inhibitors [9,10] or multitarget kinase inhibitors, including *lenvatinib* and *cabozantib* [9], whereas *omipalisib* and *dactolisib* are currently under clinical trials as agents targeting the phosphoinositide 3-kinase (PI3K) [9]. It is worth noting, however, that the antiproliferative effects of the quinoline-containing compounds may also result from cell cycle arrest [11,12,13,14,15], apoptosis [16,17], DNA intercalation [18,19], inhibition of angiogenesis [20,21,22], inhibition of proteasome [23,24], and disruption of tubulin polymerization [25,26].

**Figure 1:**
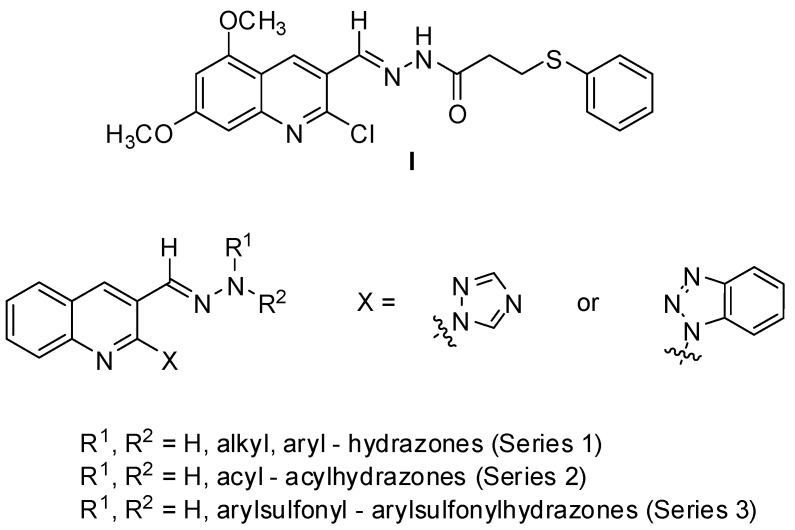
Known (**I**) and newly designed (Series 1–3) hydrazones of quinoline-3-carbaldehyde.

In this context, worth noting are anticancer quinoline-based hydrazone derivatives that have been described in review articles [27,28]. Recently, quinoline-3-carbaldehyde hydrazones of type **I** (Figure 1) have come into the focus of our research program aimed at the discovery of novel anticancer agents. As described by Bingul et al. [29], compound **I** reduced the viability of SH-SY5Y neuroblastoma cancer cells and induced G_1_ cell cycle arrest by upregulating the cell-cycle-related p27^Kip1^ protein [29].

With the above information in mind, we decided to prepare a small library of new quinoline hydrazones (series **1**) and *N*-acylhydrazones (series **2**) bearing either 1,2,4-triazole or benzotriazole at position 2 of the quinoline ring system (Figure 1) to identify compounds with potential antitumor activity. Since *N*-sulfonylhydrazones have recently been studied as antiproliferative agents [30], the arylsulfonylhydrazone group was also incorporated into the target compounds (Figure 1, series **3**).

## 2. Results and Discussion

### 2.1. Chemistry

**Scheme 1:**
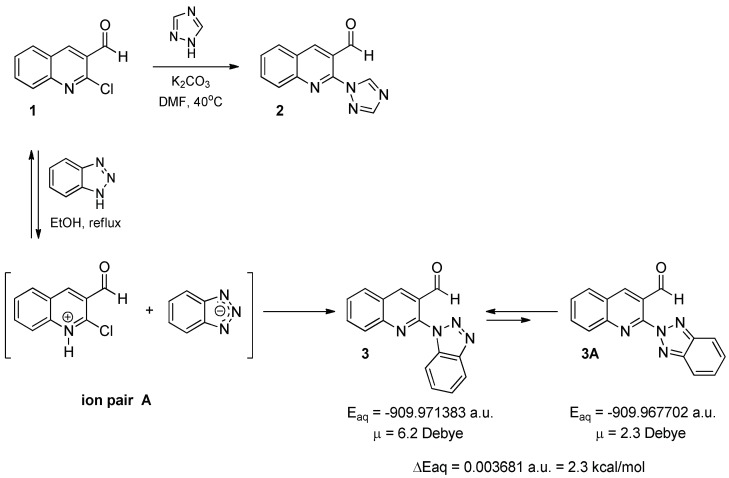
Synthesis of 2-(1*H*-1,2,4-triazol-1-yl)quinoline-3-carbaldehyde (**2**) and 2-(1*H*-benzotriazol-1-yl)quinoline-3-carbaldehyde (**3**).

Our research started with reactions of 2-chloroquinoline-3-carbaldehyde (**1**) [31,32] with 1,2,4-triazole and benzotriazole that afforded the starting 2-(1*H*-1,2,4-triazol-1-yl)quinoline-3-carbaldehyde (**2**) and 2-(1*H*-benzotriazol-1-yl)quinoline-3-carbaldehyde (**3**), respectively. As outlined in Scheme 1, the triazole-containing aldehyde **2** was obtained by heating the substrate **1** with 1,2,4-triazole in the presence of anhydrous potassium carbonate as a base. On the other hand, no base was required for preparation of the benzotriazole-containing aldehyde **3**. Apparently, benzotriazole, a stronger NH acid than triazole (p*K*a = 8.2 versus 10.3) [33], protonates the quinoline nitrogen atom which leads to the formation of ion pair **A**. Then, deprotonated benzotriazole attacks position 2 of the 2-chloroquinolinium cation to give the desired product **3** (Scheme 1).

Due to annular tautomerism of the benzotriazole ring system, the *N*-heteroarylation process may take place at either the *N*1 or *N*2 nitrogen atom depending on the reaction conditions and stereochemical properties of a product [34,35]. In our case, both proton and carbon NMR spectra of the product run in DMSO-*d*_6_ are consistent with the structure **3** (Section 3).

Structures of the benzotriazol-1-yl (**3**) and benzotriazol-2-yl (**3A**) isomers were subjected to quantum-chemical calculations by use of the density-functional B3LYP/6-31+G* method and the SM8 (H_2_O) solvation model (Scheme 1) [36]. The computations indicated that the tautomer **3** should be more stable than **3A** by 2.3 kcal/mol. Although the low energy difference suggests that both tautomers may exist in equilibrium, the *N*1-tautomer **3** with a higher dipole moment (µ = 6.2 Debye) than those found for the *N*2-tautomer **3A** (µ = 2.3 Debye) should predominate over **3A** in polar solvents. These results are consistent with the previous studies which indicated that in solution 1*H*-benzotriazole is the predominant species [37].

**Scheme 2:**
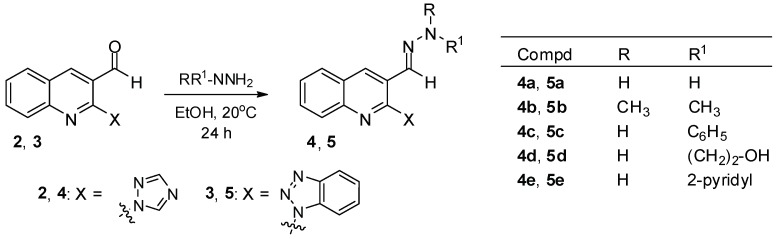
Synthesis of hydrazones **4a**–**e** and **5a**–**e** (Series 1).

Compounds **2** and **3** were then subjected to reactions with hydrazine derivatives in ethanol at ambient temperature to afford the desired hydrazones **4a**–**e** and **5a**–**e**, respectively (Series 1, Scheme 2). 

The structures of the compounds **4a**–**e** and **5a**–**e** were confirmed by elemental analyses as well as IR, NMR, and MS spectroscopic data (see Section 3).

Regarding anticancer activity of *N*-acylhydrazones, we turned our attention to the results obtained by Lima et al. [38]. It was found that the -C(O)-NH-N=C- acylhydrazone scaffold of *N*-aroylhydrazones designed as *combretastatin A4* (CA-4) analogues is bioisterically equivalent to the ethylene -CH=CH- linker. Thus, *N*-acylhydrazones comparable to *combretastatin A4* are capable of binding to the colchicine domain on β-tubulin and may prove useful in the development of new chemotherapeutic agents with better pharmacokinetic properties than the prototype CA-4.

**Scheme 3:**
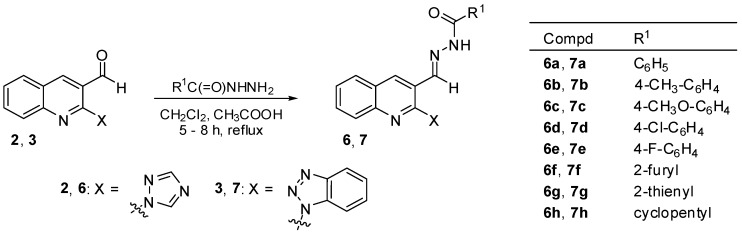
Synthesis of *N*′-acylhydrazones **6a**–**h** and **7a**–**h** (Series 2).

As shown in Scheme 3, treatment of the aldehydes **2** and **3** with the appropriate aryl- and alkylhydrazides in dichloromethane under reflux in the presence of acetic acid gave rise to the formation of the corresponding *N*′-acylhydrazones **6a**–**h** and **7a**–**h** (Series 2).

**Figure 2:**
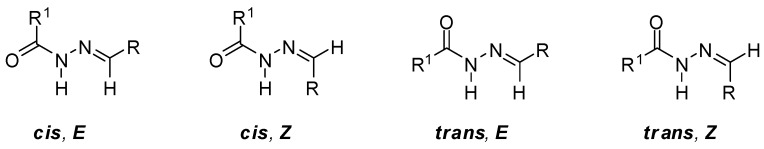
Probable *E*/*Z* isomers and *cis*/*trans* amide conformers of *N*-acyl- and *N*-aroylhydrazones.

It is well-known that *N*-acyl- and *N*-aroylhydrazones may exist as geometric isomers *E*/*Z* with respect to the C=N double bond and *cis*/*trans* amide conformers due to rotation of the amide HN–C(O) single bond (Figure 2) [39,40]. Literature reports for *N*-acyl- and *N*-aroylhydrazones derived from aryl- and heteroaryl aldehydes indicate that these compounds may exist both in DMSO-*d*_6_ solution [39,40,41,42] and solid phase [43,44,45,46] in the form of *E*-geometrical isomers. Other studies revealed the presence of the mixtures of two forms: *cis* and *trans* amide conformers of *N*-acyl- [39,40,45,46,47,48,49,50,51] and *N*-aroylhydrazones [52,53] in solution.

**Figure 3:**
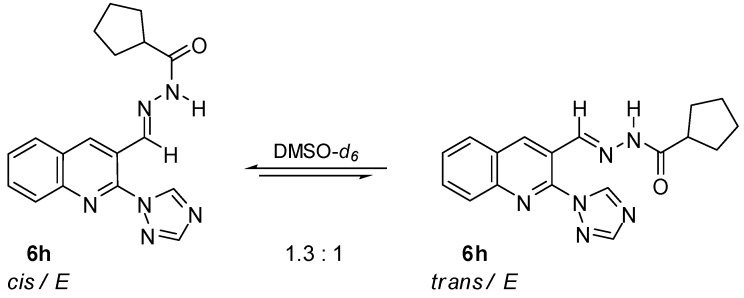
Possible isomers of *N*′-cyclopentanecarbohydrazide **6h**.

Analysis of ^1^H NMR spectra of the obtained *N*′-aroylhydrazones **6a**–**g** and **7a**–**g** run in DMSO-*d*_6_ confirmed the existence of single isomers as no duplicate signals were observed. The only exception was the *N*′-cyclopentanecarbohydrazides **6h** and **7h** that in carbon and proton NMR spectra exhibited two set of resonance signals. Following the findings of Ferreira and co-workers [50], we assumed that the observed doubled signals refer to the presence of both the *cis*/*E* and *trans*/*E* amide conformers. For example, in the ^13^C NMR spectrum of **6h**, the signals at 172.5 ppm and 177.8 ppm referred to the carbon atoms of the amide C=O group of the *cis* and *trans* conformers, while the ^1^H NMR spectrum of **6h** revealed the presence of two separate singlets at 11.67 ppm and 11.42 ppm attributable to the protons of the amide C(O)–NH group. Based on the relative intensities of these signals, we concluded that in DMSO-*d*_6_ solution the *N*′-acylhydrazone **6h** exists as a 1.3:1 mixture of equilibrating *cis*/*E* and *trans*/*E* isomers (Figure 3).

**Scheme 4:**
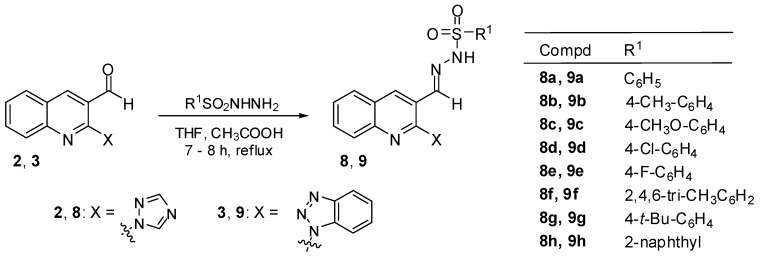
Synthesis of *N*′-sulfonylhydrazones **8a**–**h** and **9a**–**h** (Series 3).

Next, we synthesized the quinoline-3-carbaldehyde *N*′-sulfonylhydrazone derivatives **8a**–**h** and **9a**–**h** (Series 3, Scheme 4). The reactions of aldehydes **2** and **3** with arylsulfonylhydrazides proceeded smoothly in THF solution under reflux in the presence of a catalytic amount of acetic acid. The identity of the newly prepared compounds was confirmed by elemental analyses as well as the IR and NMR spectroscopic data presented in the experimental section (see Section 3).

### 2.2. UV-Vis Studies of Hydrazones **4**–**9** in Aqueous Buffer

**Figure 4:**
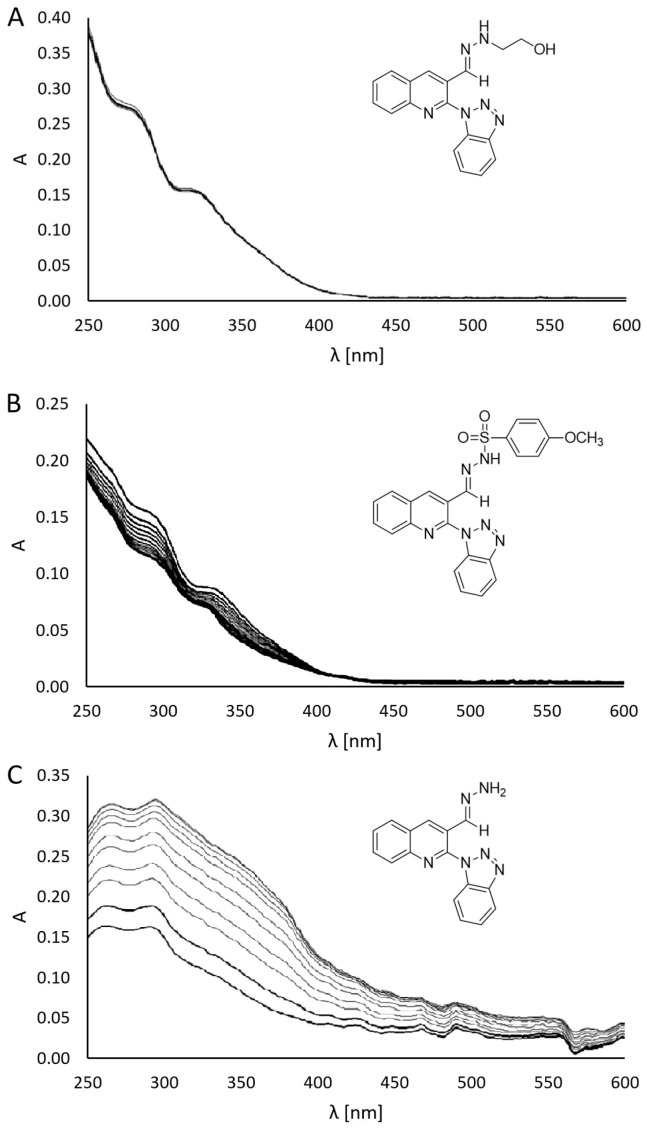
UV-Vis spectra of compounds: (**A**) **5d**; (**B**) **9c**; and (**C**) **5a** in 0.1% DMSO/PBS solution (pH 7.4) at a concentration of 40 µM and 37 °C.

The chemical stability of the hydrazones **4**–**9** in phosphate-buffered saline (PBS, pH 7.4) was investigated by means of UV-Vis spectroscopy. In general, all the compounds tested proved to be stable in the PBS solution as exemplified by the hydrazides **5a** and **5d** and the benzenesulfonohydrazide **9c**, since no new spectra with the formation of isosbestic points were observed (Figure 4). The compound **5d** (Figure 4A) showed no noticeable time-dependent changes, whereas a decrease in the intensity of the initial spectrum of the derivative **9c** (Figure 4B) is likely due to its slow precipitation out of the PBS solution.

On the other hand, the time-dependent changes in the UV-Vis spectra of the hydrazones showed that precipitation of the derivatives **4e**, **5a**, **8f**, and **9f** is rather fast as exemplified by 2-(1*H*-benzotriazol-1-yl)-3-(hydrazonomethyl)quinoline (**5a**, Figure 4C). Therefore, those poorly soluble species were excluded from a panel of compounds subjected to biological studies.

### 2.3. In Vitro Antitumor Activity

The in vitro antitumor potential of the newly synthesized quinoline-3-carbaldehyde hydrazone derivatives **4**–**9** was evaluated on three human cancer cell lines: the pancreatic cell line DAN-G, the large cell lung cancer cell line LCLC-103H, and the cervical cancer cell line SISO using a crystal violet microtiter plate assay as previously described [54]. This assay measures the antiproliferative activity of compounds on actively dividing cells. Primary screening of the compounds **4**–**9** was performed to indicate whether a substance possesses enough activity to inhibit cell growth by 50% at the concentration of 10 µM, which is a concentration attainable in cancer cells (Table 1).

As revealed by the data in Table 1, the hydrazone derivatives **4**, **6**, and **8** bearing a triazole moiety were in general inactive with the exception of the *N*-sulfonylhydrazones **8b** and **8g**, which at a concentration of 10 µM exhibited weak to moderate cytostatic effects against all investigated cancer cell lines (percent of growth in the range of 31.6–48.6%). On the other hand, replacement of the triazole ring with a benzotriazole moiety results in enhancement of activity as indicated by a comparison of the growth inhibitory activities of triazole-containing compounds with their corresponding benzotriazole ring counterparts (**6a**, **6d**, and **6f**–**g** versus **7a**, **7d**, and **7f**–**g** and **8c**–**e** and **8g**–**h** versus **9c**–**e** and **9g**–**h**). This observation may arise from the higher lipophilicity of the benzotriazole analogues, which may facilitate the penetration through the tumor cell membrane and improve the targeting efficiency. Furthermore, the combined presence of a large conjugated system as well as a three-nitrogen-containing structure make the benzotriazole nucleus more susceptible to binding with enzymes or receptors in biological systems via hydrogen bonds and π-π stacking interactions [55,56].

Thus, for secondary screening aimed at determining cytotoxic potency, we selected the benzotriazole-containing compounds **5d**–**e**, **7a**, **7d**, and **7f**–**g** and **9c**–**e** and **9g**–**h**, which demonstrated pronounced growth inhibitory effects against at least two cancer cell lines. The results of the secondary screening are presented in Table 2 as the average IC_50_ values calculated from dose-response data.

In general, the investigated compounds exhibited moderate to high growth cell inhibitory effects (IC_50_ in the range of 1.23–7.39 µM). The most potent was found to be the 2-(pyridin-2-yl)hydrazone **5e** with IC_50_ values ranging from 1.23 to 1.49 µM (Table 2). A reduction in cytotoxic potency by 2- to 6-fold was observed for other derivatives with acylhydrazone (compounds of type **7**) or sulfonylhydrazone (compounds of type **9**) moieties. However, replacing the hydrazone function with either an acylhydrazone or a sulfonylhydrazone scaffold still leads to active compounds. Hence, among the *N*′-acylhydrazones **7** and the *N*′-sulfonylhydrazones **9** the highest cytotoxic activity was found for compounds **7d** and **9d** containing a 4-chlorophenyl group (Table 2, **R^1^** = 4-ClC_6_H_4_).

It should be noted that the majority of the compounds tested showed no great selectivity toward any one specific cancer cell line with the exception of the *N*′-(benzoyl)hydrazone **7a** and the *N*′-(naphtylsulfonyl)hydrazone **9h**, which were selective against the pancreatic cell line DAN-G and the cervical cancer cell line SISO (IC_50_ values of 4.19–6.59 µM) over the lung carcinoma cell line LCLC-103H (IC_50_ >20 µM).

## 3. Materials and Methods

### 3.1. General Information

Melting points were measured on a Boetius apparatus and are uncorrected. IR spectra were taken in KBr pellets on a Nicolet 380 FTIR 1600 spectrometer. Elemental analyses were performed on a Vario El Cube CHNS analyzer and the results are within ±0.4%. NMR spectra were recorded on a Varian Gemini 200, a Varian Unity 500, or a Bruker Avance III HD apparatus. ^1^H and ^13^C chemical shifts were measured relative to the residual solvent signal at 7.26 ppm and 77.0 (CDCl_3_) or 2.50 ppm and 39.5 ppm (DMSO-*d*_6_). Coupling constants are shown in hertz (Hz). The mass spectra were recorded on a Shimadzu LCMS-2010 EV spectrometer equipped with an electrospray source. ESI-MS spectra were registered in a positive- or negative-ion mode. Preparative thin layer chromatography was performed on silica gel 60 PF_254_ containing gypsum (Merck KGaA, Darmstadt, FRG) with the aid of Chromatotron^®^ using the reported solvent systems. 2-Chloroquinoline-3-carbaldehyde (**1**) was obtained according to the published method [57]. UV-Vis spectra were recorded with an Analytik Jena Spekol 1200 (Analytik Jena AG, Jena, Germany) in a 1.0 cm cuvette maintained at 37 °C by a thermostatically controlled cuvette holder.

### 3.2. Chemistry

#### 3.2.1. Procedure for the Preparation of 2-(1*H*-1,2,4-Triazol-1-yl)quinoline-3-carbaldehyde (**2**)

To a stirred solution of 2-chloroquinoline-3-carbaldehyde (**1**) (6.0 g, 31 mmol) in DMF (20 mL), potassium carbonate (8.28 g, 60 mmol) and 1,2,4-triazole (6.32 g, 93 mmol) were added and the mixture was heated at 40 °C for 6 h. Next, the mixture was poured into crushed ice and the precipitate was collected by vacuum filtration and purified by column chromatography (silica gel) eluting with CH_2_Cl_2_/AcOEt 20:1 *v*/*v* to afford the title compound **2**. Yield 4.6 g (66%); m.p. 175–178 °C; IR (KBr) ν_max_: 3131, 3113, 3062, 2896, 1698, 1617, 1505, 1444, 1345, 1277, 1166, 1048, 984, 954, 779, 752 cm^−1^; ^1^H NMR (200 MHz, CDCl_3_) δ: 7.65–7.72 (m, 1H, Ar-H), 7.88–7.96 (m, 1H, Ar-H), 8.02–8.13 (m, 2H, Ar-H), 8.22 (s, 1H, triazole), 8.91 (s, 1H, 4-H, quinoline), 9.36 (s, 1H, triazole), 10.78 (s, 1H, CHO) ppm. Anal. calcd. for C_12_H_8_N_4_O (224.22): C, 64.28; H, 3.60; N, 24.99. Found: C, 64.39; H, 3.51; N, 24.63.

#### 3.2.2. Procedure for the Preparation of 2-(1*H*-Benzo[*d*][1,2,3]triazol-1-yl)quinoline-3-carbaldehyde (**3**)

To a stirred solution of 2-chloroquinoline-3-carbaldehyde (**1**) (6.0 g, 31 mmol) in ethanol (20 mL), benzotriazole (11.07 g, 93 mmol) in ethanol (10 mL) was added at 60 °C. After stirring at reflux for 7 h, the resulting mixture was cooled and the precipitate was collected by vacuum filtration to give the title compound **3**. Yield 7.9 g (92%); m.p. 219–221 °C; IR (KBr) ν_max_: 3056, 2922, 1690, 1618, 1583, 1498, 1446, 1286, 1161, 1070, 1024, 785, 745 cm^−1^; ^1^H NMR (200 MHz, CDCl_3_) δ: 7.56 (t, *J* = 7.9 Hz, 1H, Ar-H), 7.69–7.74 (m, 2H, Ar-H), 7.94 (t, *J* = 8.2 Hz, 1H, Ar-H), 8.08 (d, *J* = 8.1 Hz, 1H, Ar-H), 8.18–8.23 (m, 2H, Ar-H), 8.58 (d, *J* = 8.3 Hz, 1H, Ar-H), 9.00 (s, 1H, 4-H, quinoline), 10.59 (s, 1H, CHO) ppm; ^13^C NMR (200 MHz, CDCl_3_) δ: 114.6, 120.6, 124.1, 126.1, 127.0, 128.6, 129.3 (two overlapping signals), 129.8, 130.1, 133.8, 135.9, 141.8, 146.9, 148.5, 189.2 ppm; MS (ESI) *m*/*z*: 275 [M + H]^+^. Anal. calcd. for C_16_H_10_N_4_O (274.28): C, 70.06; H, 3.67; N, 20.43. Found: C, 70.18; H, 3.54; N, 20.52.

#### 3.2.3. General Procedure for the Preparation of Hydrazones **4a**–**e** and **5a**–**e**

To a suspension of 2-(1*H*-1,2,4-triazol-1-yl)quinoline-3-carbaldehyde (**2**) (0.224 g, 1 mmol) or 2-(1*H*-benzo[*d*][1,2,3]triazol-1-yl)quinoline-3-carbaldehyde (**3**) (0.274 g, 1 mmol) in ethanol (10 mL), the appropriate hydrazine (1 mmol) was added. After stirring for 24 h at ambient temperature (TLC control) the precipitated solid was collected by vacuum filtration, dried, and recrystallized or subjected to preparative thin layer chromatography. In this manner, the following compounds were obtained.

*3-(Hydrazonomethyl)-2-(1H-1,2,4-triazol-1-yl)quinoline* (**4a**). Starting from 2-(1*H*-1,2,4-triazol-1-yl)quinoline-3-carbaldehyde (**2**) (0.224 g, 1 mmol) and 67% hydrazine hydrate (1 mmol), the title compound **4a** was obtained after crystallization from ethanol. Yield 53%; m.p. 197–199 °C; IR (KBr) ν_max_: 3358, 3207, 3102, 1617, 1597, 1570, 1493, 1442, 1416, 1324, 1278, 1145, 1054, 984, 952, 785, 763, 725 cm^−1^; ^1^H NMR (500 MHz, DMSO-*d*_6_) δ: 7.39 (s, 2H, NH_2_), 7.66 (t, *J* = 7.8 Hz, 1H, Ar-H), 7.79 (t, *J* = 7.3 Hz, 1H, Ar-H), 7.84 (s, 1H, N=CH), 7.98 (d, *J* = 8.3 Hz, 1H, Ar-H), 8.13 (d, *J* = 7.8 Hz, 1H, Ar-H), 8.36 (s, 1H, triazole), 8.88 (s, 1H, 4-H, quinoline), 9.23 (s, 1H, triazole) ppm; ^13^C NMR (50 MHz, DMSO-*d*_6_) δ: 125.4, 128.6, 128.7, 128.9 (two overlapping signals), 131.2, 131.6, 134.6, 145.4, 145.9, 146.0, 153.2; MS (ESI) *m*/*z*: 239 [M + H]^+^. Anal. calcd. for C_12_H_10_N_6_ (238.25): C, 60.50; H, 4.23; N, 35.27. Found: C, 60.32; H, 4.36; N, 35.32.

*3-[(2,2-Dimethylhydrazono)methyl]-2-(1H-1,2,4-triazol-1-yl)quinoline* (**4b**). Starting from 2-(1*H*-1,2,4-triazol-1-yl)quinoline-3-carbaldehyde (**2**) (0.224 g, 1 mmol) and dimethylhydrazine (1 mmol), the title compound **4b** was obtained after preparative thin layer chromatography (eluent: CH_2_Cl_2_/AcOEt 10:1 *v*/*v*). Yield 59%; m.p. 138–141 °C; IR (KBr) ν_max_: 3129, 3048, 2925, 1618, 1551, 1501, 1493, 1438, 1402, 1279, 1142, 1070, 1045, 987, 915, 757 cm^−1^; ^1^H NMR (200 MHz, DMSO-*d*_6_) δ: 2.98 (s, 6H, 2xCH_3_), 7.44 (s, 1H, N=CH), 7.67 (t, *J* = 7.9 Hz, 1H, Ar-H), 7.81 (t, *J* = 8.3 Hz, 1H, Ar-H), 7.99 (d, *J* = 8.3 Hz, 1H, Ar-H), 8.15 (d, *J* = 7.9 Hz, 1H, Ar-H), 8.37 (s, 1H, triazole), 8.86 (s, 1H, 4-H, quinoline), 9.27 (s, 1H, triazole) ppm; ^13^C NMR (50 MHz, DMSO-*d*_6_) δ: 42.7 (two overlapping signals), 125.1 (two overlapping signals), 128.3, 128.5 (three overlapping signals), 130.7, 134.0, 144.9, 145.7 (two overlapping signals), 152.8 ppm; MS (ESI) *m*/*z*: 267 [M + H]^+^. Anal. calcd. for C_14_H_14_N_6_ (266.30): C, 63.14; H, 5.30; N, 31.56. Found: C, 63.32; H, 5.42; N, 31.26.

*3-[(2-Phenylhydrazono)methyl]-2-(1H-1,2,4-triazol-1-yl)quinoline* (**4c**). Starting from 2-(1*H*-1,2,4-triazol-1-yl)quinoline-3-carbaldehyde (**2**) (0.224 g, 1 mmol) and phenylhydrazine (1 mmol), the title compound **4c** was obtained after crystallization from toluene. Yield 32%; m.p. 182–185 °C; IR (KBr) ν_max_: 3236, 3132, 3052, 1603, 1593, 1557, 1490, 1436, 1271, 1207, 1123, 985, 959, 924, 748 cm^−1^; ^1^H NMR (500 MHz, DMSO-*d*_6_) δ: 6.80 (t, *J* = 7.3 Hz, 1H, Ar-H), 7.15 (d, *J* = 8.3 Hz, 2H, Ar-H), 7.25 (t, *J* = 7.8 Hz, 2H, Ar-H), 7.70 (t, *J* = 7.7 Hz, 1H, Ar-H), 7.82 (t, *J* = 7.3 Hz, 1H, Ar-H), 8.00 (d, *J* = 8.3 Hz, 1H, Ar-H), 8.11 (s, 1H, N=CH), 8.22 (d, *J* = 8.3 Hz, 1H, Ar-H), 8.42 (s, 1H, triazole), 9.12 (s, 1H, 4-H, quinoline), 9.29 (s, 1H, triazole), 10.82 (s, 1H, NH) ppm; ^13^C NMR (125 MHz, DMSO-*d*_6_) δ: 113.0 (two overlapping signals), 120.2, 124.6, 128.6, 128.7, 128.9, 129.1, 129.9 (two overlapping signals), 131.2, 131.5, 135.3, 145.4, 145.6, 146.0, 146.1, 153.3 ppm; MS (ESI) *m/z*: 313 [M − H]^−^. Anal. calcd. for C_18_H_14_N_6_ (314.34): C, 68.78; H, 4.49; N, 26.74. Found: C, 68.59; H, 4.38; N, 27.03.

*2-{2-[(2-(1H-1,2,4-Triazol-1-yl)quinolin-3-yl)methylene]hydrazinyl}ethanol* (**4d**). Starting from 2-(1*H*-1,2,4-triazol-1-yl)quinoline-3-carbaldehyde (**2**) (0.224 g, 1 mmol) and 2-hydrazinylethanol (1 mmol), the title compound **4d** was obtained after crystallization from ethanol. Yield 51%; m.p. 173–175 °C; IR (KBr) ν_max_: 3345, 2946, 1618, 1599, 1580, 1492, 1449, 1428, 1392, 1287, 1178, 1056, 1023, 910, 786 cm^−1^; ^1^H NMR (500 MHz, DMSO-*d*_6_) δ: 3.21–3.24 (m, 2H, CH_2_), 3.56–3.57 (m, 2H, CH_2_), 4.67 (s, 1H, OH), 7.64 (t, *J* = 7.3 Hz, 1H, Ar-H), 7.72 (s, 1H, N=CH), 7.76 (t, *J* = 7.8 Hz, 1H, Ar-H), 7.90 (t, 1H, NH), 7.96 (d, *J* = 8.3 Hz, 1H, Ar-H), 8.12 (d, *J* = 7.8 Hz, 1H, Ar-H), 8.35 (s, 1H, triazole), 8.84 (s, 1H, 4-H, quinoline), 9.23 (s, 1H, triazole) ppm; ^13^C NMR (125 MHz, DMSO-*d*_6_) δ: 51.6, 60.0, 125.6, 127.4, 128.6, 128.7, 128.8, 128.9, 130.9, 134.1, 145.2, 145.9 (two overlapping signals), 153.1 ppm; MS (ESI) *m*/*z*: 281 [M − H]^−^. Anal. calcd. for C_14_H_14_N_6_O (282.30): C, 59.56; H, 5.00; N, 29.77. Found: C, 59.38; H, 5.17; N, 29.65.

*3-[(2-(Pyridin-2-yl)hydrazono)methyl]-2-(1H-1,2,4-triazol-1-yl)quinoline* (**4e**). Starting from 2-(1*H*-1,2,4-triazol-1-yl)quinoline-3-carbaldehyde (**2**) (0.224 g, 1 mmol) and 2-hydrazinylpyridine (1 mmol), the title compound **4e** was obtained after crystallization from a DMF–methanol mixture. Yield 20%; m.p. 248–252 °C; IR (KBr) ν_max_: 3186, 3118, 3069, 3024, 1595, 1560, 1540, 1490, 1458, 1442, 1306, 1278, 1129, 1123, 991, 753 cm^−1^; ^1^H NMR (500 MHz, DMSO-*d*_6_) δ: 6.79 (t, *J* = 5.9 Hz, 1H, Ar-H), 7.32 (d, *J* = 8.3 Hz, 1H, Ar-H), 7.64–7.71 (m, 2H, Ar-H), 7.83 (t, *J* = 7.3 Hz, 1H, Ar-H), 8.01 (d, *J* = 8.3 Hz, 1H, Ar-H), 8.13 (d, *J* = 4.4 Hz, 1H, Ar-H), 8.18 (d, *J* = 8.3 Hz, 1H, Ar-H), 8.33 (s, 2H, N=CH and CH triazole), 9.07 (s, 1H, 4-H, quinoline), 9.22 (s, 1H, triazole), 10.98 (s, 1H, NH) ppm; ^13^C NMR (125 MHz, DMSO-*d*_6_) δ: 107.6, 116.2, 124.4, 128.5, 128.6, 129.0, 129.1, 131.6, 134.5, 136.4, 138.5, 145.7, 146.0, 146.4, 148.5, 153.2, 157.6 ppm. Anal. calcd. for C_17_H_13_N_7_ (315.33): C, 64.75; H, 4.16; N, 31.09. Found: C, 64.59; H, 3.98; N, 31.43.

*2-(1H-Benzo[d][1,2,3]triazol-1-yl)-3-(hydrazonomethyl)quinoline* (**5a**). Starting from 2-(1*H*-benzo[*d*][1,2,3]triazol-1-yl)quinoline-3-carbaldehyde (**3**) (0.274 g, 1 mmol) and 65% hydrazine hydrate (1 mmol), the title compound **5a** was obtained after crystallization from a DMF–methanol mixture. Yield 75%; m.p. 239–241 °C; IR (KBr) ν_max_: 3416, 3282, 3180, 3060, 1617, 1591, 1493, 1461, 1400, 1286, 1217, 1067, 1022, 1013, 928, 760, 737 cm^−1^; ^1^H NMR (200 MHz, DMSO-*d*_6_) δ: 7.39 (s, 2H, NH_2_), 7.52–7.64 (m, 2H, Ar-H), 7.66–7.91 (m, 3H, Ar-H and N=CH), 8.05 (t, 2H, Ar-H), 8.25 (t, 2H, Ar-H), 9.03 (s, 1H, 4-H, quinoline) ppm; ^13^C NMR (125 MHz, DMSO-*d*_6_) δ: 113.1, 120.1, 125.7, 126.2, 128.6, 128.8, 128.9, 129.0, 129.7, 131.3, 132.5, 133.8, 135.5, 145.8, 145.9, 146.2 ppm; MS (ESI) *m*/*z*: 289 [M + H]^+^. Anal. calcd. for C_16_H_12_N_6_ (288.31): C, 66.66; H, 4.20; N, 29.15. Found: C, 66.21; H, 4.29; N, 29.50.

*2-(1H-Benzo[d][1,2,3]triazol-1-yl)-3-[(2,2-dimethylhydrazono)methyl]quinoline* (**5b**). Starting from 2-(1*H*[*d*]-benzo[1,2,3]triazol-1-yl)quinoline-3-carbaldehyde (**3**) (0.274 g, 1 mmol) and dimethylhydrazine (1 mmol), the title compound **5b** was obtained after preparative thin layer chromatography (eluent: CH_2_Cl_2_/AcOEt 10:1 *v*/*v*). Yield 80%; m.p. 165–167 °C; IR (KBr) ν_max_: 3045, 2914, 2859, 1542, 1490, 1421, 1283, 1062, 1022, 739 cm^−1^; ^1^H NMR (200 MHz, DMSO-*d*_6_) δ: 2.91 (s, 6H, 2xCH_3_), 7.31 (s, 1H, N=CH), 7.53 (t, *J* =7.9 Hz, 1H, Ar-H), 7.70 (t, *J* = 7.9 Hz, 2H, Ar-H), 7.82 (t, *J* = 8.3 Hz, 1H, Ar-H), 8.04 (d, *J* = 8.3 Hz, 2H, Ar-H), 8.17–8.27 (m, 2H, Ar-H), 8.94 (s, 1H, 4-H, quinoline) ppm; ^13^C NMR (50 MHz, DMSO-*d*_6_) δ: 42.7 (two overlapping signals), 113.2, 119.9, 125.1, 125.4, 126.0, 128.3, 128.5, 128.6, 128.8, 129.3, 130.8, 133.3, 134.6, 145.2, 145.5, 145.9 ppm. Anal. calcd. for C_18_H_16_N_6_ (316.36): C, 68.56; H, 5.21; N, 26.23. Found: C, 68.47; H, 5.21; N, 26.32.

*2-(1H-Benzo[d][1,2,3]triazol-1-yl)-3-[(2-phenylhydrazono)methyl]quinoline* (**5c**). Starting from 2-(1*H*-benzo[*d*][1,2,3]triazol-1-yl)quinoline-3-carbaldehyde (**3**) (0.274 g, 1 mmol) and phenylhydrazine (1 mmol), the title compound **5c** was obtained after crystallization from ethanol. Yield 51%; m.p. 106–110 °C; IR (KBr) ν_max_: 3253, 3055, 1600, 1551, 1491, 1425, 1286, 1262, 1131, 1090, 1021, 1010, 929, 783, 749 cm^−1^; ^1^H NMR (500 MHz, DMSO-*d*_6_) δ: 6.78 (t, *J* = 7.3 Hz, 1H, Ar-H), 7.05 (d, *J* = 7.8 Hz, 2H, Ar-H), 7.22 (t, *J* = 7.8 Hz, 2H, Ar-H), 7.56 (t, *J* = 7.3 Hz, 1H, Ar-H), 7.69–7.76 (m, 2H, Ar-H), 7.85 (t, *J* = 7.8 Hz, 1H, Ar-H), 8.02 (s, 1H, N=CH), 8.05–8.08 (m, 2H, Ar-H), 8.27 (d, *J* = 8.8 Hz, 2H, Ar-H), 9.21 (s, 1H, 4-H, quinoline), 10.76 (s, 1H, NH) ppm; ^13^C NMR (125 MHz, DMSO-*d*_6_) δ: 113.0 (two overlapping signals), 113.5, 120.1, 120.2, 125.5, 125.9, 128.6, 128.9, 129.1 (two overlapping signals), 129.8 (three overlapping signals), 131.3, 131.6, 133.7, 135.4, 145.3, 145.8, 145.9, 146.2 ppm; MS (ESI) *m*/*z*: 363 [M − H]^−^. Anal. calcd. for C_22_H_16_N_6_ (364.40): C, 72.51; H, 4.43; N, 23.06. Found: C, 72.27; H, 4.58; N, 23.15.

*2-{2-[(2-(1H-Benzo[d][1,2,3]triazol-1-yl)quinolin-3-yl)methylene]hydrazinyl}ethanol* (**5d**). Starting from 2-(1*H*-benzo[*d*][1,2,3]triazol-1-yl)quinoline-3-carbaldehyde (**3**) (0.274 g, 1 mmol) and hydrazinylethanol (1 mmol), the title compound **5d** was obtained after crystallization from ethanol. Yield 61%; m.p. 177–179 °C; IR (KBr) ν_max_: 3344, 3139, 2934, 2877, 1596, 1571, 1509, 1493, 1441, 1328, 1284, 1211, 1141, 1071, 989, 753 cm^−1^; ^1^H NMR (200 MHz, DMSO-*d*_6_) δ: 3.15–3.19 (m, 2H, CH_2_), 3.50–3.53 (m, 2H, CH_2_), 4.64 (t, *J* = 5.3 Hz, 1H, OH), 7.55 (t, *J* = 7.8 Hz, 1H, Ar-H), 7.63 (s, 1H, N=CH), 7.66–7.70 (m, 2H, Ar-H), 7.79 (t, *J* = 7.3 Hz, 1H, Ar-H), 7.87 (t, *J* = 4.8 Hz, 1H, NH), 8.00 (d, *J* = 9.3 Hz, 2H, Ar-H), 8.18 (d, *J* = 7.8 Hz, 1H, Ar-H), 8.23 (d, *J* = 8.3 Hz, 1H, Ar-H), 8.95 (s, 1H, 4-H, quinoline) ppm; ^13^C NMR (125 MHz, DMSO-*d*_6_) δ: 51.6, 60.0, 113.3, 120.1, 125.8. 126.4, 127.4, 128.6, 128.7, 128.9, 129.0, 129.7, 131.0, 133.6, 134.6, 145.5, 145.7, 146.0 ppm; MS (ESI) *m*/*z*: 331 [M − H]^−^. Anal. calcd. for C_18_H_16_N_6_O (332.36): C, 65.05; H, 4.85; N, 25.29. Found: C, 64.89; H, 5.06; N, 25.11.

*2-(1H-Benzo[d][1,2,3]triazol-1-yl)-3-[2-(pyridin-2-yl)hydrazonomethyl]quinoline* (**5e**). Starting from 2-(1*H*-benzo[*d*][1,2,3]triazol-1-yl)quinoline-3-carbaldehyde (**3**) (0.274 g, 1 mmol) and 2-hydrazinylpyridine (1 mmol), the title compound **5e** was obtained after crystallization from a DMF/methanol mixture. Yield 54%; m.p. 266–268 °C; IR (KBr) ν_max_: 3199, 3159, 3024, 2926, 2862, 1601, 1558, 1492, 1444, 1308, 1290, 1133, 1058, 1021, 756, 738 cm^−1^; ^1^H NMR (200 MHz, DMSO-*d*_6_) δ: 6.82 (t, *J* = 6.2 Hz, 1H, Ar-H), 7.23 (d, *J* = 8.7 Hz, 1H, Ar-H), 7.57–7.82 (m, 4H, Ar-H), 7.91 (t, *J* = 6.6 Hz, 1H, Ar-H), 8.09–8.13 (m, 3H, Ar-H), 8.24 (s, 1H, N=CH), 8.30 (d, *J* = 8.3 Hz, 2H, Ar-H), 9.26 (s, 1H, 4-H, quinoline), 11.18 (s, 1H, NH) ppm; ^13^C NMR (50 MHz, DMSO-*d*_6_) δ: 107.1, 113.3, 116.0, 119.9, 124.8, 125.6, 128.2, 128.7, 128.9, 129.0, 129.5, 131.6, 133.3, 133.9, 136.5, 138.3, 145.6, 145.9, 146.2, 148.2, 157.0 ppm; MS (ESI) *m*/*z*: 364 [M − H]^−^. Anal. calcd. for C_21_H_15_N_7_ (365.39): C, 69.03; H, 4.14; N, 26.83. Found: C, 68.94; H, 3.96; N, 27.10.

#### 3.2.4. General Procedure for the Preparation of *N*′-Acylhydrazones **6a**–**h** and **7a**–**h**

A mixture of 2-(1*H*-1,2,4-triazol-1-yl)quinoline-3-carbaldehyde (**2**) (0.224 g, 1 mmol) or 2-(1*H*-benzo[*d*][1,2,3]triazol-1-yl)quinoline-3-carbaldehyde (**3**) (0.274 g, 1 mmol) and appropriate hydrazide (1 mmol) in the presence of a catalytic amount of acetic acid in dichloromethane (5 mL) was heated under reflux for 5–8 h. The progress of the reaction was controlled by TLC. The mixture was then evaporated under reduced pressure to dryness and the crude product thus obtained was purified as described below. In this manner, the following compounds were obtained.

*N′-[(2-(1H-1,2,4-Triazol-1-yl)quinolin-3-yl)methylene]benzohydrazide* (**6a**). Starting from 2-(1*H*-1,2,4-triazol-1-yl)quinoline-3-carbaldehyde (**2**) (0.224 g, 1 mmol) and benzohydrazide (1 mmol), the title compound **6a** was obtained after washing with hot ethanol. Yield 70%; m.p. 254–255 °C; IR (KBr) ν_max_: 3219, 3131, 3038, 1651, 1602, 1546, 1491, 1442, 1280, 1132, 983, 759 cm^−1^; ^1^H NMR (500 MHz, DMSO-*d*_6_) δ: 7.53 (t, *J* = 7.8 Hz, 2H, Ar-H), 7.60 (t, *J* = 7.3 Hz, 1H, Ar-H), 7.75 (t, *J* = 7.8 Hz, 1H, Ar-H), 7.90–7.93 (m, 3H, Ar-H), 8.06 (d, *J* = 8.3 Hz, 1H, Ar-H), 8.30 (d, *J* = 8.3 Hz, 1H, Ar-H), 8.43 (s, 1H, N=CH), 8.76 (s, 1H, triazole), 9.16 (s, 1H, triazole), 9.36 (s, 1H, 4-H, quinoline), 12.19 (s, 1H, NH) ppm; ^13^C NMR (125 MHz, DMSO-*d*_6_) δ: 122.7, 128.1, 128.4 (three overlapping signals), 128.9, 129.0, 129.3 (two overlapping signals), 129.6, 132.8, 133.5, 138.0, 143.9, 145.9, 146.5, 146.7, 153.5, 164.3 ppm; MS (ESI) *m*/*z*: 341 [M − H]^−^. Anal. calcd. for C_19_H_14_N_6_O (342.35): C, 66.66; H, 4.12; N, 24.55. Found: C, 66.78; H, 4.01; N, 24.38.

*N′-[(2-(1H-1,2,4-Triazol-1-yl)quinolin-3-yl)methylene]-4-methylbenzohydrazide* (**6b**). Starting from 2-(1*H*-1,2,4-triazol-1-yl)quinoline-3-carbaldehyde (**2**) (0.224 g, 1 mmol) and 4-methylbenzohydrazide (1 mmol), the title compound **6b** was obtained after washing with hot ethanol. Yield 68%; m.p. 250–254 °C; IR (KBr) ν_max_: 3243, 3081, 1663, 1559, 1507, 1492, 1444, 1275, 1209, 1118, 982, 958, 762 cm^−1^; ^1^H NMR (500 MHz, DMSO-*d*_6_) δ: 2.37 (s, 3H, CH_3_), 7.33 (d, *J* = 8.3 Hz, 2H, Ar-H), 7.74 (t, *J* = 7.3 Hz, 1H, Ar-H), 7.84 (d, *J* = 7.8 Hz, 2H, Ar-H), 7.91 (t, *J* = 7.3 Hz, 1H, Ar-H), 8.06 (d, *J* = 8.8 Hz, 1H, Ar-H), 8.30 (d, *J* = 8.3 Hz, 1H, Ar-H), 8.43 (s, 1H, N=CH), 8.76 (s, 1H, triazole), 9.14 (s, 1H, triazole), 9.36 (s, 1H, 4-H, quinoline), 12.12 (s, 1H, NH) ppm; ^13^C NMR (125 MHz, DMSO-*d*_6_) δ: 21.7, 123.1, 128.2, 128.6, 128.8, 128.9 (two overlapping signals), 129.5, 129.6 (two overlapping signals), 129.9, 131.2, 132.5, 137.9, 142.5, 145.8, 146.7, 146.8, 153.5, 164.5 ppm; MS (ESI) *m*/*z*: 355 [M − H]^−^. Anal. calcd. for C_20_H_16_N_6_O (356.38): C, 67.40; H, 4.53; N, 23.58. Found: C, 67.38; H, 4.72; N, 23.15.

*N′*-*[(2-(1H-1,2,4-Triazol-1-yl)quinolin-3-yl)methylene]-4-methoxybenzohydrazide* (**6c**). Starting from 2-(1*H*-1,2,4-triazol-1-yl)quinoline-3-carbaldehyde (**2**) (0.224 g, 1 mmol) and 4-methoxybenzohydrazide (1 mmol), the title compound **6c** was obtained after crystallization from a DMF–methanol mixture. Yield 29%; m.p. 262–263 °C; IR (KBr) ν_max_: 3216, 3132, 3029, 2958, 2835, 1646, 1602, 1508, 1440, 1254, 1174, 987, 863, 762 cm^−1^; ^1^H NMR (200 MHz, DMSO-*d*_6_) δ: 3.84 (s, 3H, OCH_3_), 7.07 (d, *J* = 8.7 Hz, 2H, Ar-H), 7.75 (t, *J* = 7.8 Hz, 1H, Ar-H), 7.89–7.96 (m, 3H, Ar-H), 8.08 (d, *J* = 8.3 Hz, 1H, Ar-H), 8.32 (d, *J* = 7.9 Hz, 1H, Ar-H), 8.44 (s, 1H, N=CH), 8.74 (s, 1H, triazole), 9.15 (s, 1H, triazole), 9.37 (s, 1H, 4-H, quinoline), 12.06 (s, 1H, NH) ppm; ^13^C NMR (125 MHz, DMSO-*d*_6_) δ: 56.1, 114.5 (two overlapping signals), 122.9, 125.5, 128.1, 128.9, 129.0, 129.5, 130.4, 132.7, 137.9, 143.2, 145.9 (two overlapping signals), 146.5, 146.7, 153.5, 162.9, 163.7 ppm; MS (ESI) *m*/*z*: 371 [M − H]^−^. Anal. calcd. for C_20_H_16_N_6_O_2_ (372.38): C, 64.51; H, 4.33; N, 22.57. Found: C, 64.41; H, 4.21; N, 22.83.

*N′*-*[(2-(1H-1,2,4-Triazol-1-yl)quinolin-3-yl)methylene]-4-chlorobenzohydrazide* (**6d**). Starting from 2-(1*H*-1,2,4-triazol-1-yl)quinoline-3-carbaldehyde (**2**) (0.224 g, 1 mmol) and 4-chlorobenzohydrazide (1 mmol), the title compound **6d** was obtained after washing with hot ethanol. Yield 21%; m.p. 272–274 °C; IR (KBr) ν_max_: 3221, 3087, 1672, 1597, 1560, 1490, 1445, 1273, 1210, 1115, 981, 958, 752 cm^−1^; ^1^H NMR (200 MHz, DMSO-*d*_6_) δ: 7.60–7.66 (m, 2H, Ar-H), 7.76 (t, *J* = 7.9 Hz, 1H, Ar-H), 7.89–7.98 (m, 3H, Ar-H), 8.06–8.10 (m, 1H, Ar-H), 8.32 (d, *J* = 7.9 Hz, 1H, Ar-H), 8.44 (s, 1H, N=CH), 8.78 (s, 1H, triazole), 9.16 (s, 1H, triazole), 9.39 (s, 1H, 4-H, quinoline), 12.24 (s, 1H, NH) ppm; ^13^C NMR (125 MHz, DMSO-*d*_6_) δ: 122.6, 128.1, 128.9, 129.0, 129.3 (two overlapping signals), 129.6, 130.4 (two overlapping signals), 132.3, 132.8, 137.6, 138.1, 144.3, 145.9, 146.6, 146.7, 153.5, 163.3 ppm; MS (ESI) *m*/*z*: 375 [M − H]^−^. Anal. calcd. for C_19_H_13_ClN_6_O (376.80): C, 60.56; H, 3.48; N, 22.30. Found: C, 60.42; H, 3.41; N, 22.17.

*N′*-*[(2-(1H-1,2,4-triazol-1-yl)quinolin-3-yl)methylene]-4-fluorobenzohydrazide* (**6e**). Starting from 2-(1*H*-1,2,4-triazol-1-yl)quinoline-3-carbaldehyde (**2**) (0.224 g, 1 mmol) and 4-fluorobenzohydrazide (1 mmol), the title compound **6e** was obtained after preparative thin layer chromatography (eluent: CH_2_Cl_2_/AcOEt 10:1 *v*/*v*). Yield 42%; m.p. 261–263 °C; IR (KBr) ν_max_: 3217, 3132, 3038, 1652, 1601, 1503, 1493, 1346, 1282, 1223, 1158, 1119, 983, 852, 759 cm^−1^; ^1^H NMR (200 MHz, DMSO-*d*_6_) δ: 7.38 (t, *J* = 8.7 Hz, 2H, Ar-H), 7.75 (t, *J* = 7.9 Hz, 1H, Ar-H), 7.88–8.09 (m, 4H, Ar-H), 8.31 (d, *J* = 7.9 Hz, 1H, Ar-H), 8.44 (s, 1H, N=CH), 8.78 (s, 1H, triazole), 9.15 (s, 1H, triazole), 9.37 (s, 1H, 4-H, quinoline), 12.21 (s, 1H, NH) ppm; ^13^C NMR (50 MHz, DMSO-*d*_6_) δ: 115.7, 116.1, 122.5, 127.9, 128.6 (two overlapping signals), 129.4, 130.0, 130.8, 131.0, 132.4, 137.6, 143.5, 145.7, 146.3, 146.5, 153.3, 162.6, 167.1 ppm; MS (ESI) *m*/*z*: 359 [M − H]^−^. Anal. calcd. for C_19_H_13_FN_6_O (360.34): C, 63.33; H, 3.64; N, 23.32. Found: C, 63.52; H, 3.51; N, 23.21.

*N′*-*[(2-(1H-1,2,4-Triazol-1-yl)quinolin-3-yl)methylene]furan-2-carbohydrazide* (**6f**). Starting from 2-(1*H*-1,2,4-triazol-1-yl)quinoline-3-carbaldehyde (**2**) (0.224 g, 1 mmol) and furan-2-carbohydrazide (1 mmol), the title compound **6f** was obtained after washing with hot ethanol. Yield 31%; m.p. 248–250 °C; IR (KBr) ν_max_: 3176, 3105, 3036, 1659, 1643, 1599, 1507, 1491, 1444, 1357, 1290, 1280, 1190, 1122, 1086, 1023, 984, 859, 783, 755 cm^−1^; ^1^H NMR (200 MHz, DMSO-*d*_6_) δ: 6.71–6.74 (m, 1H, Ar-H), 7.21–7.45 (m, 1H, Ar-H), 7.75 (t, *J* = 7.9 Hz, 1H, Ar-H), 7.89–7.98 (m, 2H, Ar-H and N=CH), 8.07 (d, *J* = 8.3 Hz, 1H, Ar-H), 8.31 (d, *J* = 8.3 Hz, 1H, Ar-H), 8.45 (s, 1H, triazole), 8.76 (br. s, 1H, Ar-H), 9.13 (s, 1H, 4-H, quinoline), 9.38 (s, 1H, triazole), 12.22 (s, 1H, NH) ppm; ^13^C NMR (50 MHz, DMSO-*d*_6_) δ: 112.6, 115.8, 122.5, 127.9, 128.6 (two overlapping signals), 129.4, 132.4, 137.6, 138.8, 143.5, 145.6, 146.3, 146.5, 146.6, 153.3, 154.3 ppm; MS (ESI) *m*/*z*: 355 [M + Na]^+^. Anal. calcd. for C_17_H_12_N_6_O_2_ (332.32): C, 61.44; H, 3.64; N, 25.29. Found: C, 61.58; H, 3.51; N, 25.34.

*N′*-*[(2-(1H-1,2,4-Triazol-1-yl)quinolin-3-yl)methylene]thiophene-2-carbohydrazide* (**6g**). Starting from 2-(1*H*-1,2,4-triazol-1-yl)quinoline-3-carbaldehyde (**2**) (0.224 g, 1 mmol) and thiophene-2-carbohydrazide (1 mmol), the title compound **6g** was obtained without further purification. Yield 36%; m.p. 265–267 °C; IR (KBr) ν_max_: 3164, 3096, 2985, 1633, 1601, 1511, 1499, 1374, 1313, 1181, 1118, 1036, 982, 757, 740 cm^−1^; ^1^H NMR (200 MHz, DMSO-*d*_6_) δ: 7.25 (t, *J* = 4.2 Hz, 1H, Ar-H), 7.76 (t, *J* = 7.1 Hz, 1H, Ar-H), 7.89–8.10 (m, 4H, Ar-H and N=CH), 8.30 (d, *J* = 7.9 Hz, 1H, Ar-H), 8.46 (s, 1H, triazole), 8.76 (br. s, 1H, Ar-H), 9.14 (s, 1H, 4-H, quinoline), 9.39 (s, 1H, triazole), 12.21 (br. s, 1H, NH) ppm; ^13^C NMR (50 MHz, DMSO-*d*_6_) δ: 122.4, 126.5, 127.8, 128.6 (two overlapping signals), 129.4, 129.9, 132.4, 135.7, 137.7, 140.5, 143.0, 145.7, 146.2, 146.5, 153.3, 158.0 ppm, MS (ESI) *m*/*z*: 347 [M − H]^−^. Anal. calcd. for C_17_H_12_N_6_OS (348.38): C, 58.61; H, 3.47; N, 24.12. Found: C, 58.49; H, 3.35; N, 24.23.

*N′*-*[(2-(1H-1,2,4-Triazol-1-yl)quinolin-3-yl)methylene]cyclopentanecarbohydrazide* (**6h**). Starting from 2-(1*H*-1,2,4-triazol-1-yl)quinoline-3-carbaldehyde (**2**) (0.224 g, 1 mmol) and cyclopentanecarbohydrazide (1 mmol), the title compound **6h** was obtained as a mixture of *cis*/*trans* conformers after preparative thin layer chromatography (eluent: CH_2_Cl_2_:AcOEt 10:1 *v*/*v*). Yield 42%; m.p. 239–241 °C; IR (KBr) ν_max_: 3196, 3103, 2959, 2866, 1661, 1599, 1491, 1442, 1395, 1257, 1140, 1124, 982, 950, 760 cm^−1^; ^1^H NMR (200 MHz, DMSO-*d*_6_) δ: 1.66–1.98 (m, 16H, 8xCH_2_), 2.62–2.69 and 3.53–4.21 (m, 1H, CH), 7.73 (t, *J* = 7.5 Hz, 2H, 2xCH), 7.90 (t, *J* = 8.3 Hz, 2H, 2xCH), 8.03–8.07 (m, 2H, 2xCH), 8.24–8.31 (m, 3H, 3xCH), 8.40 and 8.44 (s, 1H, triazole), 8.51 (s, 1H, N=CH), 9.02 and 9.06 (s, 1H, 4-H, quinoline), 9.32 and 9.36 (s, 1H, triazole), 11.42 and 11.67 (s, 1H, NH) ppm; ^13^C NMR (50 MHz, DMSO-*d*_6_) δ: 26.2, 26.3, 29.8, 30.4, 43.5, 127.8, 128.5, 129.2, 132.3, 137.3, 137.4, 138.4, 141.4, 145.5, 145.6, 153.1, 153.3, 172.5 and 177.8 ppm; MS (ESI) *m*/*z*: 333 [M − H]^−^. Anal. calcd. for C_18_H_18_N_6_O (334.38): C, 64.66; H, 5.43; N, 25.13. Found: C, 64.72; H, 5.51; N, 25.17.

*N′-[(2-(1H-Benzo[d][1,2,3]triazol-1-yl)quinolin-3-yl)methylene]benzohydrazide* (**7a**). Starting from 2-(1*H*-benzo[*d*][1,2,3]triazol-1-yl)quinoline-3-carbaldehyde (**3**) (0.274 g, 1 mmol) and benzohydrazide (1 mmol), the title compound **7a** was obtained after crystallization from methanol. Yield 77%; m.p. 236–239 °C; IR (KBr) ν_max_: 3234, 3047, 1654, 1545, 1491, 1378, 1283, 1068, 1017, 784, 748, 739 cm^−1^; ^1^H NMR (500 MHz, DMSO-*d*_6_) δ: 7.51 (t, *J* = 7.8 Hz, 2H, Ar-H), 7.57–7.61 (m, 2H, Ar-H), 7.73–7.80 (m, 2H, Ar-H), 7.90–7.95 (m, 3H, Ar-H), 8.12 (d, *J* = 8.3 Hz, 1H, Ar-H), 8.23 (d, *J* = 8.3 Hz, 1H, Ar-H), 8.27 (d, *J* = 8.3 Hz, 1H, Ar-H), 8.36 (d, *J* = 7.8 Hz, 1H, Ar-H), 8.75 (s, 1H, N=CH), 9.26 (s, 1H, 4-H, quinoline), 12.17 (s, 1H, NH) ppm; ^13^C NMR (125 MHz, DMSO-*d*_6_) δ: 114.1, 120.2, 123.6, 126.2, 128.0, 128.4 (two overlapping signals), 129.0, 129.1, 129.2 (two overlapping signals), 129.7, 130.0 (two overlapping signals), 132.7, 133.4, 133.8, 138.2, 143.8, 146.0, 146.8, 147.2, 164.0 ppm; MS (ESI) *m*/*z*: 391 [M − H]^−^. Anal. calcd. for C_23_H_16_N_6_O (392.41): C, 70.40; H, 4.11; N, 21.42. Found: C, 70.52; H, 4.21; N, 21.67.

*N′-[2-(1H-Benzo[d][1,2,3]triazol-1-yl)quinolin-3-yl)methylene]-4-methylbenzohydrazide (***7b***).* Starting from 2-(1*H*-benzo[*d*][1,2,3]triazol-1-yl)quinoline-3-carbaldehyde (**3**) (0.274 g, 1 mmol) and 4-methylbenzohydrazide (1 mmol), the title compound **7b** was obtained after crystallization from *n*-butyl alcohol. Yield 40%; m.p. 226–230 °C; IR (KBr) ν_max_: 3233, 3047, 2922, 1655, 1546, 1491, 1462, 1444, 1378, 1284, 1068, 1018, 749 cm^−1^; ^1^H NMR (500 MHz, DMSO-*d*_6_) δ: 2.37 (s, 3H, CH_3_), 7.32 (d, *J* = 8.3 Hz, 2H, Ar-H), 7.61 (t, *J* = 7.8 Hz, 1H, Ar-H), 7.74–7.79 (m, 2H, Ar-H), 7.82 (d, *J* = 8.3 Hz, 2H, Ar-H), 7.95 (t, *J* = 8.3 Hz, 1H, Ar-H), 8.13 (d, *J* = 8.3 Hz, 1H, Ar-H), 8.23 (d, *J* = 8.3 Hz, 1H, Ar-H), 8.28 (d, *J* = 8.3 Hz, 1H, Ar-H), 8.37 (d, *J* = 8.3 Hz, 1H, Ar-H), 8.74 (s, 1H, N=CH), 9.27 (s, 1H, 4-H, quinoline), 12.09 (s, 1H, NH) ppm; MS (ESI) *m*/*z*: 405 [M − H]^−^. Anal. calcd. for C_24_H_18_N_6_O (406.44): C, 70.92; H, 4.46; N, 20.68. Found: C, 70.86; H, 4.62; N, 20.55.

*N′-[2-(1H-Benzo[d][1,2,3]triazol-1-yl)quinolin-3-yl)methylene]-4-methoxybenzohydrazide* (**7c**). Starting from 2-(1*H*-benzo[*d*][1,2,3]triazol-1-yl)quinoline-3-carbaldehyde (**3**) (0.274 g, 1 mmol) and 4-methoxybenzohydrazide (1 mmol), the title compound **7c** was obtained after crystallization from *n*-butyl alcohol. Yield 35%; m.p. 242–245 °C; IR (KBr) ν_max_: 3217, 3043, 2989, 2964, 2835, 1647, 1603, 1544, 1490, 1460, 1368, 1288, 1262, 1070, 1016, 784 cm^−1^; ^1^H NMR (500 MHz, DMSO-*d*_6_) δ: 3.82 (s, 3H, OCH_3_), 7.04 (d, *J* = 8.8 Hz, 2H, Ar-H), 7.60 (t, *J* = 7.8 Hz, 1H, Ar-H), 7.73–7.80 (m, 2H, Ar-H), 7.90 (d, *J* = 8.8 Hz, 2H, Ar-H), 7.92–7.95 (m, 1H, Ar-H), 8.12 (d, *J* = 8.3 Hz, 1H, Ar-H), 8.22 (d, *J* = 8.3 Hz, 1H, Ar-H), 8.28 (d, *J* = 8.3 Hz, 1H, Ar-H), 8.35 (d, *J* = 7.8 Hz, 1H, Ar-H), 8.73 (s, 1H, N=CH), 9.25 (s, 1H, 4-H, quinoline), 12.04 (s, 1H, NH) ppm; ^13^C NMR (50 MHz, DMSO-*d*_6_) δ: 55.9, 113.7, 114.1 (three overlapping signals), 119.9, 123.5, 125.5, 125.9, 127.7, 128.7 (two overlapping signals), 129.4, 129.7, 130.1, 132.4, 133.2, 137.8, 142.8, 145.6, 146.5, 146.9, 162.6, 163.0 ppm; MS (ESI) *m*/*z*: 421 [M − H]^−^. Anal. calcd. for C_24_H_18_N_6_O_2_ (422.44): C, 68.24; H, 4.29; N, 19.89. Found: C, 68.43; H, 4.18; N, 19.60.

*N′-[2-(1H-Benzo[d][1,2,3]triazol-1-yl)quinolin-3-yl)methylene]-4-chlorobenzohydrazide* (**7d**). Starting from 2-(1*H*-benzo[*d*][1,2,3]triazol-1-yl)quinoline-3-carbaldehyde (**3**) (0.274 g, 1 mmol) and 4-chlorobenzohydrazide (1 mmol), the title compound **7d** was obtained after crystallization from *n*-butyl alcohol. Yield 80%; m.p. 238–240 °C; IR (KBr) ν_max_: 3234, 3067, 1656, 1593, 1547, 1490, 1462, 1375, 1297, 1282, 1066, 1017, 785, 748 cm^−1^; ^1^H NMR (200 MHz, CDCl_3_) δ: 7.26–7.43 (m, 3H, Ar-H), 7.50–7.67 (m, 2H, Ar-H), 7.72–7.88 (m, 3H, Ar-H), 7.96–8.09 (m, 3H, Ar-H), 8.24 (d, *J* = 8.0 Hz, 1H, Ar-H), 9.00 (s, 1H, N=CH), 9.22 (s, 1H, 4-H, quinoline), 10.62 (s, 1H, NH) ppm; ^13^C NMR (125 MHz, DMSO-*d*_6_) δ: 114.1, 120.2, 123.5, 126.2, 128.0, 129.0, 129.1, 129.3 (two overlapping signals), 129.7, 130.0, 130.4 (two overlapping signals), 132.5, 132.7, 133.4, 137.5, 138.2, 144.2, 145.9, 146.8, 147.2, 162.9 ppm; MS (ESI) *m*/*z*: 425 [M − H]^−^. Anal. calcd. for C_23_H_15_ClN_6_O (426.86): C, 64.72; H, 3.54; N, 19.69. Found: C, 64.46; H, 3.89; N, 20.00.

*N′-[2-(1H-Benzo[d][1,2,3]triazol-1-yl)quinolin-3-yl)methylene]-4-fluorobenzohydrazide* (**7e**). Starting from 2-(1*H*-benzo[*d*][1,2,3]triazol-1-yl)quinoline-3-carbaldehyde (**3**) (0.274 g, 1 mmol) and 4-fluorobenzohydrazide (1 mmol), the title compound **7e** was obtained after preparative thin layer chromatography (eluent: CH_2_Cl_2_/AcOEt 10:1 *v*/*v*). Yield 61%; m.p. 256–258 °C; IR (KBr) ν_max_: 3202, 3066, 2924, 1655, 1600, 1555, 1505, 1491, 1462, 1378, 1288, 1228, 1067, 1018, 748 cm^−1^; ^1^H NMR (200 MHz, DMSO-*d*_6_) δ: 7.37 (t, *J* = 8.3 Hz, 2H, Ar-H), 7.61 (t, *J* = 8.3 Hz, 1H, Ar-H), 7.72–7.83 (m, 2H, Ar-H), 7.97–8.04 (m, 3H, Ar-H), 8.14 (d, *J* = 8.3 Hz, 1H, Ar-H), 8.24–8.38 (m, 3H, Ar-H), 8.76 (s, 1H, N=CH), 9.27 (s, 1H, 4-H, quinoline), 12.19 (s, 1H, NH) ppm; ^13^C NMR (50 MHz, DMSO-*d*_6_) δ: 113.8, 115.7, 116.1, 119.9, 123.3, 125.9, 127.7, 128.8 (two overlapping signals), 129.4, 129.7, 129.9, 130.8, 131.0, 132.4, 133.1, 137.9, 143.6, 145.7, 146.5, 146.9, 162.6, 167.1 ppm; MS (ESI) *m*/*z*: 409 [M − H]^−^. Anal. calcd. for C_23_H_15_FN_6_O (410.40): C, 67.31; H, 3.68; N, 20.48. Found: C, 67.23; H, 3.47; N, 20.27.

*N′-[2-(1H-Benzo[d][1,2,3]triazol-1-yl)quinolin-3-yl)methylene]furan-2-carbohydrazide* (**7f**). Starting from 2-(1*H*-benzo[*d*][1,2,3]triazol-1-yl)quinoline-3-carbaldehyde (**3**) (0.274 g, 1 mmol) and furan-2-carbohydrazide (1 mmol), the title compound **7f** was obtained after washing with hot ethanol. Yield 53%; m.p. 283–285 °C; IR (KBr) ν_max_: 3254, 3147, 3072, 1663, 1597, 1565, 1544, 1492, 1467, 1301, 1200, 1069, 1017, 784, 751 cm^−1^; ^1^H NMR (500 MHz, DMSO-*d*_6_) δ: 6.69–6.70 (m, 1H, Ar-H), 7.28–7.34 (m, 1H, Ar-H), 7.60 (t, *J* = 7.3 Hz, 1H, Ar-H), 7.72–7.78 (m, 2H, Ar-CH), 7.91–7.94 (m, 2H, Ar-H and N=CH), 8.11 (d, *J* = 8.3 Hz, 1H, Ar-H), 8.22 (d, *J* = 8.3 Hz, 1H, Ar-H), 8.27 (d, *J* = 8.3 Hz, 1H, Ar-H), 8.34 (d, *J* = 8.3 Hz, 1H, Ar-H), 8.74 (s, 1H, Ar-H), 9.22 (s, 1H, 4-H, quinoline), 12.18 (s, 1H, NH) ppm; ^13^C NMR (125 MHz, DMSO-*d*_6_) δ: 112.9, 114.0, 116.1, 120.2, 123.5, 126.1, 128.0, 129.0, 129.1, 129.7, 130.0 (two overlapping signals), 132.7, 133.4, 138.2, 143.9, 146.0, 146.8, 147.1, 147.2, 155.0 ppm; MS (ESI) *m*/*z*: 381 [M − H]^−^. Anal. calcd. for C_21_H_14_N_6_O_2_ (382.37): C, 65.96; H, 3.69; N, 21.98. Found: C, 65.87; H, 3.75; N, 22.11.

*N′-[2-(1H-Benzo[d][1,2,3]triazol-1-yl)quinolin-3-yl)methylene]thiophene-2-carbohydrazide* (**7g**). Starting from 2-(1*H*-benzo[*d*][1,2,3]triazol-1-yl)quinoline-3-carbaldehyde (**3**) (0.274 g, 1 mmol) and thiophene-2-carbohydrazide (1 mmol), the title compound **7g** was obtained after washing with hot ethanol. Yield 53%; m.p. 237–239 °C; IR (KBr) ν_max_: 3243, 3106, 1647, 1596, 1548, 1492, 1426, 1381, 1284, 1064, 1015, 784, 750 cm^−1^; ^1^H NMR (500 MHz, DMSO-*d*_6_) δ: 7.18–7.24 (m, 1H, Ar-H), 7.59 (t, *J* = 7.8 Hz, 1H, Ar-H), 7.73–7.84 (m, 2H, Ar-H), 7.89–7.97 (m, 3H, Ar-H), 8.12 (d, *J* = 8.8 Hz, 1H, Ar-H), 8.24–8.28 (m, 2H, Ar-H and N=CH), 8.33 (d, *J* = 7.8 Hz, 1H, Ar-H), 8.73 (br. s, 1H, Ar-H), 9.22 (s, 1H, 4-H, quinoline), 12.17 (s, 1H, NH) ppm; ^13^C NMR (50 MHz, DMSO-*d*_6_) δ: 113.9, 119.9 (two overlapping signals), 123.2, 125.9 (two overlapping signals), 127.6, 128.7 (two overlapping signals), 129.4, 129.7 (two overlapping signals), 132.4, 133.1, 135.5, 138.0, 138.5, 143.2, 145.7, 146.5, 146.9 ppm; MS (ESI) *m*/*z*: 397 [M − H]^−^. Anal. calcd. for C_21_H_14_N_6_OS (398.44): C, 63.30; H, 3.54; N, 21.09. Found: C, 63.15; H, 3.27; N, 21.44.

*N′-[2-(1H-Benzo[d][1,2,3]triazol-1-yl)quinolin-3-yl)methylene]cyclopentanecarbohydrazide* (**7h**). Starting from 2-(1*H*-benzo[*d*][1,2,3]triazol-1-yl)quinoline-3-carbaldehyde (**3**) (0.274 g, 1 mmol) and cyclopentanecarbohydrazide (1 mmol), the title compound **7h** was obtained as a mixture of *cis*/*trans* conformers after preparative thin layer chromatography (eluent: CH_2_Cl_2_: AcOEt 10:1 *v*/*v*). Yield 71%; m.p. 202–204 °C; IR (KBr) ν_max_: 3199, 3057, 2954, 2867, 1665, 1619, 1560, 1493, 1463, 1447, 1384, 1288, 1214, 1062, 1020, 784, 747 cm^−1^; ^1^H NMR (200 MHz, DMSO-*d*_6_) δ: 1.60–1.68 (m, 16H, 8xCH_2_), 2.60–2.63 and 3.19–3.42 (m, 1H, CH), 7.48–7.62 (m, 2H, 2xCH), 7.65–7.82 (m, 4H, 4xCH), 7.94 (t, *J* = 7.9 Hz, 2H, 2xCH), 8.11–8.15 (m, 3H, 3xCH), 8.27–8.32 (m, 6H, 6xCH), 8.48 (s, 1H, N=CH), 9.11 and 9.18 (s, 1H, 4-H, quinoline), 11.36 and 11.64 (s, 1H, NH) ppm; ^13^C NMR (50 MHz, DMSO-*d*_6_) δ: 26.2 (two overlapping signals), 29.7, 30.4, 43.6, 113.8, 119.9, 125.9, 127.7, 128.7, 129.2, 129.5, 129.7, 132.3, 133.1, 137.8, 138.2, 138.6, 141.6, 145.6, 146.4, 172.5 and 177.7 ppm; MS (ESI) *m*/*z*: 383 [M − H]^−^. Anal. calcd. for C_22_H_20_N_6_O (384.43): C, 68.73; H, 5.24; N, 21.86. Found: C, 68.82; H, 5.32; N, 21.47.

#### 3.2.5. General Procedure for the Preparation of *N*′-Sulfonylhydrazones **8a**–**h** and **9a**–**h**

A mixture of 2-(1*H*-1,2,4-triazol-1-yl)quinoline-3-carbaldehyde (**2**) (0.224 g, 1 mmol) or 2-(1*H*-benzo[*d*][1,2,3]triazol-1-yl)quinoline-3-carbaldehyde (**3**) (0.274 g, 1 mmol) and appropriate sulfonohydrazide (1 mmol) in the presence of a catalytic amount of acetic acid in THF (5 mL) was heated under reflux for 7–8 h. The progress of the reaction was controlled by TLC. The mixture was then evaporated under reduced pressure and the crude product thus obtained was purified as described below. In this manner, the following compounds were obtained.

*N′-[(2-(1H-1,2,4-Triazol-1-yl)quinolin-3-yl)methylene]benzenesulfonohydrazide* (**8a**). Starting from 2-(1*H*-1,2,4-triazol-1-yl)quinoline-3-carbaldehyde (**2**) (0.224 g, 1 mmol) and benzenesulfonohydrazide (1 mmol), the title compound **8a** was obtained after washing with hot methanol. Yield 35%; m.p. 193–196 °C; IR (KBr) ν_max_: 3115, 3072, 2978, 2910, 1620, 1605, 1566, 1511, 1495, 1442, 1338, 1284, 1164, 1063, 1048, 938, 895, 760 cm^−1^; ^1^H NMR (200 MHz, DMSO-*d*_6_) δ: 7.62 (d, *J* = 8.3 Hz, 2H, Ar-H), 7.76 (t, *J* = 7.9 Hz, 1H, Ar-H), 7.90–8.00 (m, 4H, Ar-H), 8.06–8.10 (m, 1H, Ar-H), 8.33 (d, *J* = 7.9 Hz, 1H, Ar-H), 8.45 (s, 1H, N=CH), 8.78 (s, 1H, triazole), 9.17 (s, 1H, 4-H, quinoline), 9.38 (s, 1H, triazole), 12.25 (s, 1H, NH) ppm; ^13^C NMR (125 MHz, DMSO-*d*_6_) δ: 121.8, 127.8 (three overlapping signals), 128.8, 128.9, 129.5, 130.1 (two overlapping signals), 132.8, 134.0, 137.7, 139.6, 143.4, 145.8, 146.3, 146.4, 153.4 ppm. Anal. calcd. for C_18_H_14_N_6_O_2_S (378.41): C, 57.13; H, 3.73; N, 22.21. Found: C, 56.83; H, 3.65; N, 22.65.

*N′-[(2-(1H-1,2,4-Triazol-1-yl)quinolin-3-yl)methylene]-4-methylbenzenesulfonohydrazide* (**8b**). Starting from 2-(1*H*-1,2,4-triazol-1-yl)quinoline-3-carbaldehyde (**2**) (0.224 g, 1 mmol) and 4-methylbenzenesulfonohydrazide (1 mmol), the title compound **8b** was obtained after washing with hot methanol. Yield 44%; m.p. 180–186 °C; IR (KBr) ν_max_: 3110, 2916, 1620, 1599, 1512, 1493, 1441, 1345, 1284, 1165, 1067, 951, 902, 759 cm^−1^; ^1^H NMR (200 MHz, DMSO-*d*_6_) δ: 2.36 (s, 3H, CH_3_), 7.44 (d, *J* = 7.9 Hz, 2H, Ar-H), 7.75 (t, *J* = 7.1 Hz, 1H, Ar-H), 7.77–7.93 (m, 3H, Ar-H), 8.03 (d, *J* = 7.1 Hz, 1H, Ar-H), 8.25–8.30 (m, 2H, Ar-H and N=CH), 8.38 (s, 1H, triazole), 8.87 (s, 1H, 4-H, quinoline), 9.30 (s, 1H, triazole), 11.38 (s, 1H, NH) ppm; ^13^C NMR (125 MHz, DMSO-*d*_6_) δ: 21.7, 121.9, 127.8, 127.9 (two overlapping signals), 128.8, 128.9, 129.5, 130.5 (two overlapping signals), 132.8, 136.7, 137.6, 143.2, 144.6, 145.8, 146.3, 146.4, 153.4 ppm; MS (ESI) *m*/*z*: 415 [M + Na]^+^. Anal. calcd. for C_19_H_16_N_6_O_2_S (392.43): C, 58.15; H, 4.11; N, 21.42. Found: C, 57.93; H, 3.95; N, 21.71.

*N′-[(2-(1H-1,2,4-Triazol-1-yl)quinolin-3-yl)methylene]-4-methoxybenzenesulfonohydrazide* (**8c**). Starting from 2-(1*H*-1,2,4-triazol-1-yl)quinoline-3-carbaldehyde (**2**) (0.224 g, 1 mmol) and 4-methoxybenzenesulfonohydrazide (1 mmol), the title compound **8c** was obtained after washing with hot methanol. Yield 52%; m.p. 185–188 °C; IR (KBr) ν_max_: 3110, 3058, 2882, 2799, 1618, 1567, 1512, 1490, 1441, 1338, 1282, 1176, 1167, 1065, 992, 944, 787, 762 cm^−1^; ^1^H NMR (200 MHz, DMSO-*d*_6_) δ: 3.82 (s, 3H, OCH_3_), 7.15 (d, *J* = 9.1 Hz, 2H, Ar-H), 7.74 (t, *J* = 7.1 Hz, 1H, Ar-H), 7.85–7.94 (m, 3H, Ar-H), 8.01–8.06 (m, 1H, Ar-H), 8.25–8.30 (m, 2H, Ar-H and N=CH), 8.38 (s, 1H, triazole), 8.87 (s, 1H, 4-H, quinoline), 9.30 (s, 1H, triazole), 11.74 (s, 1H, NH) ppm; ^13^C NMR (125 MHz, DMSO-*d*_6_) δ: 56.4, 115.2 (two overlapping signals), 121.9, 127.9, 128.8, 128.9, 129.5, 130.2 (two overlapping signals), 131.1, 132.8, 137.6, 143.0, 145.8, 146.3, 146.4, 153.4, 163.4 ppm. Anal. calcd. for C_19_H_16_N_6_O_3_S (408.43): C, 55.87; H, 3.95; N, 20.58. Found: C, 55.97; H, 4.15; N, 20.58.

*N′-[(2-(1H-1,2,4-Triazol-1-yl)quinolin-3-yl)methylene]-4-chlorobenzenesulfonohydrazide* (**8d**). Starting from 2-(1*H*-1,2,4-triazol-1-yl)quinoline-3-carbaldehyde (**2**) (0.224 g, 1 mmol) and 4-chlorobenzenesulfonohydrazide (1 mmol), the title compound **8d** was obtained after washing with hot methanol. Yield 71%; m.p. 196–200 °C; IR (KBr) ν_max_: 3110, 3058, 2882, 2799, 1618, 1567, 1512, 1490, 1441, 1338, 1282, 1176, 1167, 1065, 944, 787, 762 cm^−1^; ^1^H NMR (200 MHz, DMSO-*d*_6_) δ: 7.69–7.73 (m, 3H, Ar-H), 7.87–7.90 (m, 1H, Ar-H), 7.94 (d, *J* = 8.8 Hz, 2H, Ar-H), 8.01–8.02 (m, 1H, Ar-H), 8.22 (d, *J* = 8.3 Hz, 1H, Ar-H), 8.34 (s, 1H, triazole), 8.36 (s, 1H, N=CH), 8.84 (s, 1H, 4-H, quinoline), 9.27 (s, 1H, triazole), 11.89 (s, 1H, NH) ppm; ^13^C NMR (125 MHz, DMSO-*d*_6_) δ: 121.9, 127.9, 128.8, 128.9, 129.0, 129.6 (two overlapping signals), 130.2 (two overlapping signals), 132.7, 137.8, 138.7, 138.8, 144.0, 145.8, 146.4, 146.6, 153.5 ppm. Anal. calcd. for C_18_H_13_ClN_6_O_2_S (412.85): C, 52.37; H, 3.17; N, 20.36. Found: C, 52.45; H, 3.37; N, 20.14.

*N′-[(2-(1H-1,2,4-Triazol-1-yl)quinolin-3-yl)methylene]-4-fluorobenzenesulfonohydrazide* (**8e**). Starting from 2-(1*H*-1,2,4-triazol-1-yl)quinoline-3-carbaldehyde (**2**) (0.224 g, 1 mmol) and 4-fluorobenzenesulfonohydrazide (1 mmol), the title compound **8e** was obtained after crystallization from *n*-butyl alcohol. Yield 38%; m.p. 184–186 °C; IR (KBr) ν_max_: 3110, 3066, 2909, 2799, 1619, 1592, 1511, 1492, 1441, 1330, 1284, 1231, 1171, 1066, 944, 832, 757 cm^−1^; ^1^H NMR (500 MHz, DMSO-*d*_6_) δ: 7.48 (t, *J* = 8.8 Hz, 2H, Ar-H), 7.71 (t, *J* = 7.8 Hz, 1H, Ar-H), 7.87 (t, *J* = 7.8 Hz, 1H, Ar-H), 8.00–8.02 (m, 3H, Ar-H), 8.24 (d, *J* = 8.3 Hz, 1H, Ar-H), 8.32 (s, 1H, N=CH), 8.37 (s, 1H, triazole), 8.85 (s, 1H, 4-H, quinoline), 9.30 (s, 1H, triazole), 11.94 (s, 1H, NH) ppm; ^13^C NMR (125 MHz, DMSO-*d*_6_) δ: 117.2, 117.4, 121.8, 127.9, 128.8, 129.6, 131.0, 131.1, 132.7, 136.0, 137.7, 143.8, 145.8, 146.4, 146.5, 153.3, 164.3, 166.3 ppm; MS (ESI) *m*/*z*: 397 [M + H]^+^. Anal. calcd. for C_18_H_13_FN_6_O_2_S (396.40): C, 54.54; H, 3.31; N, 21.20. Found: C, 54.37; H, 3.18; N, 21.58.

*N′-[(2-(1H-1,2,4-Triazol-1-yl)quinolin-3-yl)methylene]-2,4,6-trimethylbenzenesulfonohydrazide* (**8f**). Starting from 2-(1*H*-1,2,4-triazol-1-yl)quinoline-3-carbaldehyde (**2**) (0.224 g, 1 mmol) and 2,4,6-trimethylbenzenesulfonohydrazide (1 mmol), the title compound **8f** was obtained after crystallization from *n*-butyl alcohol. Yield 65%; m.p. 184–186 °C; IR (KBr) ν_max_: 3115, 3071, 2909, 1604, 1511, 1495, 1442, 1338, 1284, 1164, 1063, 1048, 938, 895, 760 cm^−1^; ^1^H NMR (500 MHz, DMSO-*d*_6_) δ: 2.21 (s, 3H, CH_3_), 2.64 (s, 6H, 2xCH_3_), 7.04 (s, 2H, Ar-H), 7.69 (t, *J* = 7.3 Hz, 1H, Ar-H), 7.85 (t, *J* = 7.3 Hz, 1H, Ar-H), 7.99 (d, *J* = 8.3 Hz, 1H, Ar-H), 8.10 (d, *J* = 8.3 Hz, 1H, Ar-H), 8.28 (s, 1H, N=CH), 8.35 (s, 1H, triazole), 8.65 (s, 1H, 4-H, quinoline), 9.26 (s, 1H, triazole), 11.97 (s, 1H, NH) ppm; ^13^C NMR (125 MHz, DMSO-*d*_6_) δ: 21.0, 23.2 (two overlapping signals), 122.3, 127.9, 128.9 (two overlapping signals), 129.2 (two overlapping signals), 132.3 (three overlapping signals), 134.4, 137.2, 139.9, 141.4, 143.1, 145.6, 146.3, 146.5, 153.3 ppm. Anal. calcd. for C_21_H_20_N_6_O_2_S (420.49): C, 59.98; H, 4.79; N, 19.99. Found: C, 60.13; H, 4.87; N, 19.67.

*N′-[(2-(1H-1,2,4-Triazol-1-yl)quinolin-3-yl)methylene]-4-tert-butylbenzenesulfonohydrazide* (**8g**). Starting from 2-(1*H*-1,2,4-triazol-1-yl)quinoline-3-carbaldehyde (**2**) (0.224 g, 1 mmol) and 4-*tert*-butylbenzenesulfonohydrazide (1 mmol), the title compound **8g** was obtained after washing with hot methanol. Yield 35%; m.p. 142–146 °C; IR (KBr) ν_max_: 3113, 3067, 2962, 2798, 1619, 1597, 1566, 1491, 1440, 1338, 1283, 1168, 1066, 944, 787, 761 cm^−1^; ^1^H NMR (200 MHz, DMSO-*d*_6_) δ: 1.27 (s, 9H, 3xCH_3_), 7.63–7.71 (m, 3H, Ar-H), 7.85–7.90 (m, 3H, Ar-H), 8.01–8.05 (m, 1H, Ar-H), 8.25–8.38 (m, 3H, Ar-H, N=CH and CH-triazole), 8.88 (s, 1H, 4-H, quinoline), 9.30 (s, 1H, triazole), 11.88 (s, 1H, NH) ppm; ^13^C NMR (125 MHz, DMSO-*d*_6_) δ: 31.4 (three overlapping signals), 35.6, 121.8, 127.0 (two overlapping signals), 127.8 (two overlapping signals), 127.9, 128.8, 129.9, 129.5, 132.8, 136.8, 137.6, 143.1, 145.8, 146.3, 146.4, 153.4, 157.0 ppm; MS (ESI) *m*/*z*: 457 [M + Na]^+^. Anal. calcd. for C_22_H_22_N_6_O_2_S (434.51): C, 60.81; H, 5.10; N, 19.34. Found: C, 60.63; H, 4.98; N, 19.66.

*N′-[(2-(1H-1,2,4-Triazol-1-yl)quinolin-3-yl)methylene]naphthalene-2-sulfonohydrazide* (**8h**). Starting from 2-(1*H*-1,2,4-triazol-1-yl)quinoline-3-carbaldehyde (**2**) (0.224 g, 1 mmol) and naphthalene-2-sulfonohydrazide (1 mmol), the title compound **8h** was obtained after washing with hot methanol. Yield 59%; m.p. 183–187 °C; IR (KBr) ν_max_: 3111, 3056, 2907, 2795, 1619, 1602, 1511, 1492, 1441, 1338, 1283, 1165, 1066, 954, 812, 747 cm^−1^; ^1^H NMR (500 MHz, DMSO-*d*_6_) δ: 7.65–7.71 (m, 3H, Ar-H), 7.86 (t, *J* = 8.3 Hz, 1H, Ar-H), 7.95 (dd, *J* = 8.8 Hz, *J* = 1.5 Hz, 1H, Ar-H), 7.97–8.02 (m, 2H, Ar-H), 8.15 (d, *J* = 8.8 Hz, 1H, Ar-H), 8.22 (d, *J* = 7.8 Hz, 1H, Ar-H), 8.27 (d, *J* = 8.8 Hz, 1H, Ar-H), 8.31 (s, 1H, N=CH), 8.35 (s, 1H, triazole), 8.67 (s, 1H, Ar-H), 8.87 (s, 1H, 4-H, quinoline), 9.27 (s, 1H, triazole), 11.97 (s, 1H, NH) ppm; ^13^C NMR (125 MHz, DMSO-*d*_6_) δ: 106.3, 121.8, 123.2, 127.9, 128.4, 128.6, 128.8, 129.2, 129.5, 129.8, 130.0, 130.1, 132.5, 132.7, 135.1, 136.7, 137.6, 143.4, 145.8, 146.4, 146.5, 153.5 ppm. Anal. calcd. for C_22_H_16_N_6_O_2_S (428.47): C, 61.67; H, 3.76; N, 19.61. Found: C, 61.43; H, 3.67; N, 19.30.

*N′-[(2-1H-Benzo[d][1,2,3]triazol-1-yl)quinolin-3-yl)methylene]benzenesulfonohydrazide* (**9a**). Starting from 2-(1*H*-benzo[*d*][1,2,3]triazol-1-yl)quinoline-3-carbaldehyde (**3**) (0.274 g, 1 mmol) and benzenesulfonohydrazide (1 mmol), the title compound **9a** was obtained after washing with methanol. Yield 53%; m.p. 118–121 °C; IR (KBr) ν_max_: 3101, 3059, 2923, 2853, 1621, 1603, 1496, 1449, 1363, 1324, 1286, 1164, 1091, 1065, 947, 750 cm^−1^; ^1^H NMR (400 MHz, DMSO-*d*_6_) δ: 7.58–7.68 (m, 4H, Ar-H), 7.70–7.80 (m, 2H, Ar-H), 7.94–7.96 (m, 3H, Ar-H), 8.12 (d, *J* = 8.3 Hz, 1H, Ar-H), 8.22–8.26 (m, 2H, Ar-H), 8.28 (s, 1H, N=CH), 8.33 (d, *J* = 8.1 Hz, 1H, Ar-H), 8.98 (s, 1H, 4-H, quinoline), 11.90 (s, 1H, NH) ppm. MS (ESI): *m*/*z*: 451 [M + Na]^+^. Anal. calcd. for C_22_H_16_N_6_O_2_S (428.47): C, 61.67; H, 3.76; N, 19.61. Found: C, 61.87; H, 3.98; N, 19.29.

*N′-[(2-1H-Benzo[d][1,2,3]triazol-1-yl)quinolin-3-yl)methylene]-4-methylbenzenesulfonohydrazide* (**9b**). Starting from 2-(1*H*-benzo[*d*][1,2,3]triazol-1-yl)quinoline-3-carbaldehyde (**3**) (0.274 g, 1 mmol) and 4-methylbenzenesulfonohydrazide (1 mmol), the title compound **9b** was obtained after preparative thin layer chromatography (eluent: CH_2_Cl_2_: AcOEt 10:1 *v*/*v*). Yield 41%; m.p. 193–197 °C; IR (KBr) ν_max_: 3214, 3064, 2955, 2855, 2772, 1597, 1149, 1447, 1370, 1324, 1286, 1163, 1049, 1020, 943, 763 cm^−1^; ^1^H NMR (500 MHz, DMSO-*d*_6_) δ: 2.35 (s, 3H, CH_3_), 7.42 (d, *J* = 7.8 Hz, 2H, Ar-H), 7.58 (t, *J* = 8.3 Hz, 1H, Ar-H), 7.71 (t, *J* = 7.8 Hz, 1H, Ar-H), 7.76 (t, *J* = 7.8 Hz, 1H, Ar-H), 7.79 (d, *J* = 8.3 Hz, 2H, Ar-H), 7.92 (t, *J* = 8.3 Hz, 1H, Ar-H), 8.09 (d, *J* = 8.3 Hz, 1H, Ar-H), 8.20 (d, *J* = 8.3 Hz, 1H, Ar-H), 8.24–8.25 (m, 2H, Ar-H and N=CH), 8.31 (d, *J* = 8.3 Hz, 1H, Ar-H), 8.96 (s, 1H, 4-H, quinoline), 11.80 (s, 1H, NH) ppm; ^13^C NMR (125 MHz, DMSO-*d*_6_) δ: 21.6, 113.9, 120.1, 122.9, 126.1, 127.7, 127.8 (two overlapping signals), 129.0 (two overlapping signals), 129.4, 129.9, 130.4 (two overlapping signals), 132.7, 133.4, 137.0, 138.0 (two overlapping signals), 143.3, 144.3, 145.9, 146.8 ppm. MS (ESI): *m*/*z*: 441 [M − H]^−^. Anal. calcd. for C_23_H_18_N_6_O_2_S (442.49): C, 62.43; H, 4.10; N, 18.99. Found: C, 62.27; H, 3.98; N, 19.35.

*N′-[(2-1H-Benzo[d][1,2,3]triazol-1-yl)quinolin-3-yl)methylene]-4-methoxybenzenesulfonohydrazide* (**9c**). Starting from 2-(1*H*-benzo[*d*][1,2,3]triazol-1-yl)quinoline-3-carbaldehyde (**3**) (0.274 g, 1 mmol) and 4-methoxybenzenesulfonohydrazide (1 mmol), the title compound **9c** was obtained after washing with hot methanol. Yield 63%; m.p. 198–202 °C; IR (KBr) ν_max_: 3149, 3069, 2860, 2760, 1595, 1578, 1493, 1426, 1352, 1290, 1264, 1163, 1022, 953, 785, 745 cm^−1^; ^1^H NMR (500 MHz, DMSO-*d*_6_) δ: 3.81 (s, 3H, OCH_3_), 7.13 (d, *J* = 8.8 Hz, 2H, Ar-H), 7.58 (t, *J* = 7.8 Hz, 1H, Ar-H), 7.72 (t, *J* = 7.8 Hz, 1H, Ar-H), 7.76 (t, *J* = 7.8 Hz, 1H, Ar-H), 7.85 (d, *J* = 8.8 Hz, 2H, Ar-H), 7.92 (t, *J* = 7.8 Hz, 1H, Ar-H), 8.10 (d, *J* = 8.3 Hz, 1H, Ar-H), 8.20 (d, *J* = 8.3 Hz, 1H, Ar-H), 8.23–8.26 (m, 2H, Ar-H and N=CH), 8.31 (d, *J* = 8.3 Hz, 1H, Ar-H), 8.96 (s, 1H, 4-H, quinoline), 11.71 (s, 1H, NH) ppm; ^13^C NMR (125 MHz, DMSO-*d*_6_) δ: 56.4, 114.2, 115.2 (two overlapping signals), 120.2, 122.9, 126.1, 127.7, 129.0, 129.1, 129.6, 129.9, 130.1 (two overlapping signals), 131.3, 132.7, 133.3, 137.9, 143.1, 145.9, 146.7, 146.9, 163.4 ppm; MS (ESI): *m*/*z*: 457 [M − H]^−^. Anal. calcd. for C_23_H_18_N_6_O_3_S (458.49): C, 60.25; H, 3.96; N, 18.33. Found: C, 60.12; H, 3.76; N, 18.65.

*N′-[(2-1H-Benzo[d][1,2,3]triazol-1-yl)quinolin-3-yl)methylene]-4-chlorobenzenesulfonohydrazide* (**9d**). Starting from 2-(1*H*-benzo[*d*][1,2,3]triazol-1-yl)quinoline-3-carbaldehyde (**3**) (0.274 g, 1 mmol) and 4-chlorobenzenesulfonohydrazide (1 mmol), the title compound **9d** was obtained after washing with hot methanol. Yield 45%; m.p. 202–206 °C; IR (KBr) ν_max_: 3190, 3064, 2875, 1598, 1587, 1494, 1431, 1355, 1320, 1173, 1091, 1067, 1022, 952, 785, 757 cm^−1^; ^1^H NMR (500 MHz, DMSO-*d*_6_) δ: 7.57 (t, *J* = 7.3 Hz, 1H, Ar-H), 7.66–7.74 (m, 3H, Ar-H), 7.75 (t, *J* = 7.3 Hz, 1H, Ar-H), 7.77–7.93 (m, 3H, Ar-H), 8.09 (d, *J* = 8.3 Hz, 1H, Ar-H), 8.19 (d, *J* = 8.3 Hz, 1H, Ar-H), 8.22 (d, *J* = 8.3 Hz, 1H, Ar-H), 8.26 (d, *J* = 7.8 Hz, 1H, Ar-H), 8.32 (s, 1H, N=CH), 8.93 (s, 1H, 4-H, quinoline), 11.78 (s, 1H, NH) ppm; ^13^C NMR (125 MHz, DMSO-*d*_6_) δ: 114.1, 120.2, 122.8, 126.0, 127.8, 128.9, 129.0, 129.5, 129.7 (two overlapping signals), 129.8 (two overlapping signals), 130.1 (two overlapping signals), 132.7, 133.4, 138.2, 138.8, 144.1, 146.0, 146.9, 147.0 ppm. Anal. calcd. for C_22_H_15_ClN_6_O_2_S (462.91): C, 57.08; H, 3.27; N, 18.15. Found: C, 56.87; H, 3.15; N, 18.54.

*N′-[(2-1H-Benzo[d][1,2,3]triazol-1-yl)quinolin-3-yl)methylene]-4-fluorobenzenesulfonohydrazide (***9e***).* Starting from 2-(1*H*-benzo[*d*][1,2,3]triazol-1-yl)quinoline-3-carbaldehyde (**3**) (0.274 g, 1 mmol) and 4-fluorobenzenesulfonohydrazide (1 mmol), the title compound **9e** was obtained after washing with hot methanol. Yield 56%; m.p. 205–209 °C; IR (KBr) ν_max_: 3071, 2869, 2771, 1618, 1590, 1493, 1424, 1326, 1289, 1238, 1170, 1056, 1024, 941, 838, 757, 748 cm^−1^; ^1^H NMR (500 MHz, DMSO-*d*_6_) δ: 7.45 (t, *J* = 8.8 Hz, 2H, Ar-H), 7.57 (t, *J* = 7.8 Hz, 1H, Ar-H), 7.71 (t, *J* = 7.3 Hz, 1H, Ar-H), 7.75 (t, *J* = 7.3 Hz, 1H, Ar-H), 7.92 (t, *J* = 7.8 Hz, 1H, Ar-H), 7.97–8.00 (m, 2H, Ar-H), 8.09 (d, *J* = 8.3 Hz, 1H, Ar-H), 8.20 (d, *J* = 8.3 Hz, 1H, Ar-H), 8.23 (d, *J* = 8.3 Hz, 1H, Ar-H), 8.27–8.29 (m, 2H, Ar-H and N=CH), 8.94 (s, 1H, 4-H, quinoline), 11.82 (s, 1H, NH) ppm; ^13^C NMR (125 MHz, DMSO-*d*_6_) δ: 114.2, 117.1, 117.3, 120.2, 122.8, 126.1, 127.7, 128.9, 129.0, 129.6, 129.9, 131.0, 132.7, 133.3, 136.2, 138.2, 144.0, 146.0, 146.8, 147.0, 164.2, 166.2 ppm. Anal. calcd. for C_22_H_15_FN_6_O_2_S (446.46): C, 59.18; H, 3.39; N, 18.82. Found: C, 59.38; H, 3.16; N, 18.56.

*N*′-*[(2-1H-Benzo[d][1,2,3]triazol-1-yl)quinolin-3-yl)methylene]-2,4,6-trimethylbenzenesulfonohydrazide* (**9f**). Starting from 2-(1*H*-benzo[*d*][1,2,3]triazol-1-yl)quinoline-3-carbaldehyde (**3**) (0.274 g, 1 mmol) and 2,4,6-trimethylbenzenesulfonohydrazide (1 mmol), the title compound **9f** was obtained after washing with hot methanol. Yield 57%; m.p. 187–191 °C; IR (KBr) ν_max_: 3213, 3060, 2938, 1601, 1493, 1448, 1424, 1316, 1164, 1052, 1022, 941, 889, 750 cm^−1^; ^1^H NMR (500 MHz, DMSO-*d*_6_) δ: 2.23 (s, 3H, CH_3_), 2.64 (s, 6H, 2xCH_3_), 7.05 (s, 2H, Ar-H), 7.58 (t, *J* = 7.8 Hz, 1H, Ar-H), 7.70–7.76 (m, 2H, Ar-H), 7.91 (t, *J* = 8.3 Hz, 1H, Ar-H), 8.08 (d, *J* = 8.3 Hz, 1H, Ar-H), 8.17–8.25 (m, 3H, Ar-H), 8.26 (s, 1H, N=CH), 8.80 (s, 1H, 4-H, quinoline), 11.92 (s, 1H, NH) ppm; ^13^C NMR (125 MHz, DMSO-*d*_6_) δ: 21.1, 23.4 (two overlapping signals), 114.2, 120.2, 123.0, 126.1, 127.6, 128.9, 129.0, 129.5, 129.9, 132.4 (three overlapping signals), 132.6, 133.2, 134.1, 137.4, 139.8, 141.6, 143.1, 145.9, 146.6, 146.9 ppm. Anal. calcd. for C_25_H_22_N_6_O_2_S (470.55): C, 63.81; H, 4.71; N, 17.86. Found: C, 63.61; H, 4.58; N, 17.58.

*N′-[(2-1H-Benzo[d][1,2,3]triazol-1-yl)quinolin-3-yl)methylene]-4-tert-butylbenzenesulfonohydrazide* (**9g**). Starting from 2-(1*H*-benzo[*d*][1,2,3]triazol-1-yl)quinoline-3-carbaldehyde (**3**) (0.274 g, 1 mmol) and 4-*tert*-butylbenzenesulfonohydrazide (1 mmol), the title compound **9g** was obtained after washing with hot methanol. Yield 57%; m.p. 132–136 °C; IR (KBr) ν_max_: 3187, 3061, 2965, 2870, 2771, 1593, 1495, 1463, 1429, 1357, 1321, 1290, 1164, 1064, 1027, 944, 783 cm^−1^; ^1^H NMR (200 MHz, DMSO-*d*_6_) δ: 1.26 (s, 9H, 3xCH_3_), 7.58 (t, *J* = 7.8 Hz, 1H, Ar-H), 7.64 (d, *J* = 8.8 Hz, 2H, Ar-H), 7.72 (t, *J* = 7.8 Hz, 1H, Ar-H), 7.77 (t, *J* = 7.8 Hz, 1H, Ar-H), 7.84 (d, *J* = 8.8 Hz, 2H, Ar-H), 7.92 (t, *J* = 8.3 Hz, 1H, Ar-H), 8.10 (d, *J* = 8.3 Hz, 1H, Ar-H), 8.21 (d, *J* = 8.3 Hz, 1H, Ar-H), 8.25–8.26 (m, 2H, Ar-H and N=CH), 8.31 (d, *J* = 7.8 Hz, 1H, Ar-H), 8.98 (s, 1H, 4-H, quinoline), 11.85 (s, 1H, NH) ppm; ^13^C NMR (125 MHz, DMSO-*d*_6_) δ: 31.4 (three overlapping signals), 35.5, 113.9, 120.1, 123.0, 126.0, 126.7 (two overlapping signals), 127.7 (two overlapping signals), 127.8, 129.0 (two overlapping signals), 129.4, 129.9 (two overlapping signals), 132.7, 133.4, 137.1, 138.1, 143.3, 146.0, 146.9, 157.1 ppm; MS (ESI): *m*/*z*: 483 [M − H]^−^. Anal. calcd. for C_26_H_24_N_6_O_2_S (484.57): C, 64.44; H, 4.99; N, 17.34. Found: C, 64.34; H, 4.78; N, 17.71.

*N′-[(2-1H-Benzo[d][1,2,3]triazol-1-yl)quinolin-3-yl)methylene]naphthalene-2-sulfonohydrazide (***9h***).* Starting from 2-(1*H*-benzo[*d*][1,2,3]triazol-1-yl)quinoline-3-carbaldehyde (**3**) (0.274 g, 1 mmol) and naphthalene-2-sulfonohydrazide (1 mmol), the title compound **9h** was obtained after washing with hot methanol. Yield 52%; m.p. 196–200 °C; IR (KBr) ν_max_: 3179, 3055, 2915, 1618, 1587, 1493, 1446, 1427, 1328, 1289, 1164, 1051, 1021, 958, 783, 752 cm^−1^; ^1^H NMR (500 MHz, DMSO-*d*_6_) δ: 7.56 (t, *J* = 7.3 Hz, 1H, Ar-H), 7.66–7.71 (m, 3H, Ar-H), 7.75 (t, *J* = 7.8 Hz, 1H, Ar-H), 7.89–7.94 (m, 2H, Ar-H), 8.02 (d, *J* = 7.8 Hz, 1H, Ar-H), 8.07 (d, *J* = 8.3 Hz, 1H, Ar-H), 8.14–8.19 (m, 2H, Ar-H), 8.23 (d, *J* = 8.3 Hz, 1H, Ar-H), 8.26–8.29 (m, 3H, Ar-H and N=CH), 8.65 (s, 1H, Ar-H), 8.98 (s, 1H, 4-H, quinoline), 11.95 (s, 1H, NH) ppm; ^13^C NMR (125 MHz, DMSO-*d*_6_) δ: 113.9, 120.1, 122.9, 123.1, 126.0, 127.7, 128.4, 128.5, 129.9 (two overlapping signals), 129.0, 129.4, 129.7, 129.8 (two overlapping signals), 129.9, 130.0, 132.5, 132.7, 133.4, 135.2, 136.9, 138.2, 143.6, 146.0, 146.8 ppm. Anal. calcd. for C_26_H_18_N_6_O_2_S (478.53): C, 65.26; H, 3.79; N, 17.56. Found: C, 65.17; H, 3.58; N, 17.96.

### 3.3. Stability Studies

To 5.0 mL of PBS (phosphate-buffered saline, pH 7.4), pre-warmed at 37 °C, was added 10 µL of a 20 mM DMSO solution of the quinoline derivative, resulting in a final compound concentration of 40 µM. The solution was then transferred to a 1.0 cm quartz cuvette and placed in a heated cuvette holder maintained at 37 °C. Spectra were recorded at 10 min intervals between wavelengths of 250 and 600 nm by means of an Analytik Jena Spekol 1200 (Analytik Jena AG) diode array UV-Vis spectrophotometer connected to a personal computer (PC) running the Aspect Plus (V 1.5) software (Analytik Jena AG).

### 3.4. In Vitro Cytotoxicity Studies

All cell culture reagents were purchased from Sigma (Deisenhofen, Germany). The cancer cell lines human pancreas cell adenocarcinoma DAN-G, human large cell lung carcinoma LCLC-103H, and human uterine cervical adenocarcinoma SISO were obtained from the German Collection of Microorganisms and Cell Cultures (DSMZ, Braunschweig, Germany, FRG). The culture medium for cell lines was RPMI-1640 medium containing 2 g/L HCO_3_ and 10% fetal calf serum (FCS). Cells were grown in 75 cm^2^ plastic culture flasks (Sarstedt, Nümbrecht, Germany, FRG) in a humid atmosphere of 5% CO_2_ at 37 °C and were passaged shortly before becoming confluent. Cytotoxicity studies were performed with a well-established microtiter assay based on the staining of adherent cells with crystal violet and performed as previously described [54]. Briefly, a volume of 100 µL of a cell suspension were seeded into 96-well microtiter plates (Sarstedt) at a density of 1000 cells per well except for the LCLC-103H cell line, which was plated out at 250 cells per well. Twenty-four hours later, cells were exposed to the substance at five concentrations per compound. The 1000-fold concentrated stock solutions in DMSO were serially diluted by 50% in DMSO to give the feed solutions, which were diluted 500-fold into culture medium. The controls received just DMSO. Each concentrate was tested in eight wells, with each well receiving 100 µL of the medium containing the substance. The concentration ranges were chosen to bracket the expected IC_50_ values as best as possible. Cells were then incubated for 48 h, after which time the medium was removed and replaced with 1% glutaraldehyde/PBS. The cells were then stained with crystal violet and the optical density (OD) was measured at λ = 570 nm with an Anthos 2010 plate reader (Salzburg, Austria).

The corrected percent growth values [*T*/*C_corr_*(%)] were calculated by the equation:

*T*/*C_corr_*(%) = (OD_T_ − OD_c,0_)/(OD_c_ − OD_c,0_) × 100

where OD_T_ is the mean absorbance of the treated cells, OD_c_ is the mean absorbance of the controls, and OD_c,0_ is the mean absorbance at the time the drug was added. The IC_50_ values were estimated by a linear least-squares regression of the *T*/*C_corr_* values versus the logarithm of the substance concentration; only concentrations that yielded *T*/*C_corr_* values between 10% and 90% were used in the calculation. The reported IC_50_ values are the averages of three independent experiments.

## 4. Conclusions

**Figure 5:**
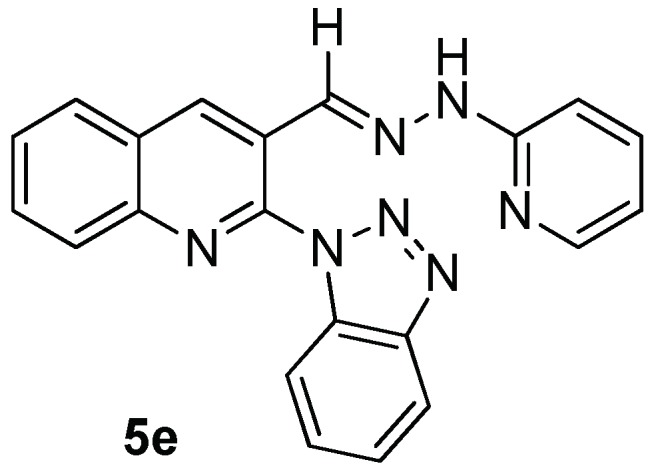
Structure of 2-(1*H*-benzo[*d*][1,2,3]triazol-1-yl)-3-[(2-(pyridin-2-yl)hydrazonomethyl]-quinoline **5e**.

In this study, we have investigated the anticancer properties of three series of quinoline-3-carbaldehyde hydrazone derivatives possessing either 1,2,4-triazole or 1,2,3-benzotriazole rings. Analysis of the structure-activity relationships of cytotoxic activities on the human cancer cell lines of 1,2,4-triazole-containing quinolines **4**, **6**, and **8** and 1,2,3-benzotriazole-containing quinolines **5**, **7**, and **9** revealed that the less lipophilic 1,2,4-triazole derivatives are generally inactive, while the more lipophilic 1,2,3-benzotriazole analogues exhibit moderate to high cytotoxic effects. It is too early to speculate on the mechanism of action of these compounds. Nonetheless, the most active 2-(1*H*-benzo[*d*][1,2,3]triazol-1-yl)-3-[(2-(pyridin-2-yl)hydrazonomethyl]-quinoline (Series 1, compound **5e,** Figure 5) with IC_50_ values in the range of 1.23–1.49 µM may serve as a useful lead compound in the development of new chemotherapeutic agents.

## Figures and Tables

**Table 1:**
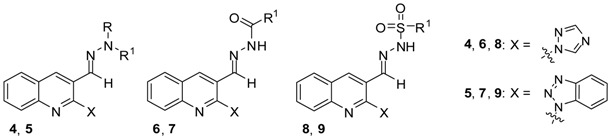
Percent of cell growth (%) relative to untreated control after 48 h at a concentration of 10 µM *.

No.	R	R^1^	Cell Line		
			DAN-G	LCLC-103H	SISO
**4a** **	H	H	87.4 ± 18.1	82.7 ± 31.9	85.3 ± 10.4
**4b**	CH_3_	CH_3_	79.6 ± 0.1	69.2 ± 0.1	70.7 ± 0.1
**4c**	H	C_6_H_5_	104.8 ± 0.1	118.9 ± 0.1	105.5 ± 0.1
**4d**	H	(CH_2_)_2_-OH	82.6 ± 0.1	63.4 ± 0.1	75.6 ± 0.1
**5b**	CH_3_	CH_3_	100.7 ± 8.2	94.2 ± 0.1	100.5 ± 7.1
**5c**	H	C_6_H_5_	54.8 ± 24.8	54.2 ± 17.5	63.5 ± 20.7
**5d**	H	(CH_2_)_2_-OH	47.6 ± 13.6	18.8 ± 21.6	36.0 ± 21.0
**5e**	H	2-pyridyl	31.8 ± 27.1	−1.3 ± 0.1	2.9 ± 3.8
**6a**	−	C_6_H_5_	72.0 ± 18.7	55.5 ± 31.1	81.7 ± 18.3
**6b**	−	4-CH_3_-C_6_H_4_	90.0 ± 5.1	89.2 ± 0.9	99.4 ± 2.4
**6c**	−	4-CH_3_O-C_6_H_4_	86.1 ± 2.5	94.7 ± 2.1	91.1 ± 2.2
**6d**	−	4-Cl-C_6_H_4_	83.2 ± 13.2	90.9 ± 3.47	91.8 ± 1.9
**6e**	−	4-F-C_6_H_4_	88.4 ± 4.7	91.4 ± 9.3	91.5 ± 1.9
**6f**		2-furyl	81.9 ± 17.0	91.4 ± 0.6	89.5 ± 0.5
**6g**	−	2-thienyl	57.5 ± 4.0	63.3 ± 28.7	65.8 ± 0.5
**6h**	−	cyclopentyl	56.1 ± 5.0	55.1 ± 23.7	62.1 ± 0.8
**7a**	−	C_6_H_5_	14.1 ± 14.5	30.0 ± 11.8	10.0 ± 9.0
**7b**	−	4-CH_3_-C_6_H_4_	42.4 ± 19.1	38.5 ± 16.9	26.5 ± 19.3
**7c**	−	4-CH_3_O-C_6_H_4_	56.3 ± 20.5	43.6 ± 15.9	53.8 ± 14.9
**7d**	−	4-Cl-C_6_H_4_	12.3 ± 7.1	−3.6 ± 4.4	6.1 ± 3.0
**7e**	−	4-F-C_6_H_4_	53.2 ± 17.8	9.0 ± 5.8	52.4 ± 8.6
**7f**		2-furyl	24.8 ± 31.3	30.4 ± 37.9	26.1 ± 9.8
**7g**	−	2-thienyl	0.6 ± 4.3	14.4 ± 19.6	0.2 ± 4.0
**7h**	−	cyclopentyl	35.3 ± 15.9	51.1 ± 12.4	32.5 ± 16.3
**8a** **	−	C_6_H_5_	82.5 ± 18.2	73.4 ± 26.6	81.4 ± 14.4
**8b** **	−	4-CH_3_-C_6_H_4_	35.6 ± 3.4	31.6 ± 3.8	33.0 ± 0.2
**8c**	−	4-CH_3_O-C_6_H_4_	62.6 ± 0.1	83.6 ± 0.2	74.1 ± 0.1
**8d**	−	4-Cl-C_6_H_4_	73.7 ± 0.1	89.6 ± 0.1	78.4 ± 0.1
**8e**	−	4-F-C_6_H_4_	78.0 ± 0.1	94.9 ± 0.2	75.4 ± 0.1
**8g** **	−	4-*t*-Bu-C_6_H_4_	39.8 ± 6.1	39.8 ± 2.1	46.8 ± 9.7
**8h**	−	2-naphthyl	88.0 ± 0.1	117.9 ± 0.1	102.9 ± 0.1
**9a**	−	C_6_H_5_	56.6 ± 20.1	15.2 ± 16.2	34.4 ± 25.5
**9b**	−	4-CH_3_-C_6_H_4_	82.1 ± 69.1	31.1 ± 25.8	24.8 ± 12.6
**9c**	−	4-CH_3_O-C_6_H_4_	35.4 ± 34.9	11.3 ± 16.2	4.1 ± 5.8
**9d**	−	4-Cl-C_6_H_4_	30.7 ± 34.9	8.9 ± 6.0	0.6 ± 0.7
**9e**	−	4-F-C_6_H_4_	66.1 ± 60.6	37.5 ± 34.2	13.2 ± 5.3
**9g**	−	4-*t*-Bu-C_6_H_4_	26.1 ± 22.4	8.1 ± 1.4	5.1 ± 6.1
**9h**	−	2-naphthyl	85.1 ± 73.7	37.5 ± 32.9	19.2 ± 11.5

* Valus are averages of three or ** two independent determinations with standard deviations.

**Table 2:**
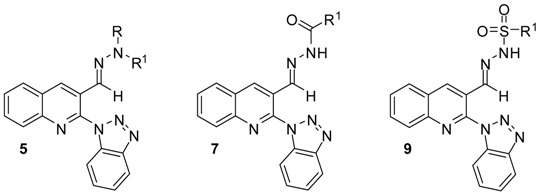
IC_50_ (µM) values in three human cancer cell lines obtained after 48 h exposure *.

No.	R	R^1^	Cell Line		
			DAN-G	LCLC-103H	SISO
**5d**	H	(CH_2_)_2_-OH	6.38 ± 1.80	6.29 ± 1.99	6.23 ± 1.46
**5e**	H	2-pyridyl	1.35 ± 0.54	1.23 ± 0.74	1.49 ± 0.23
**7a**	−	C_6_H_5_	6.35 ± 1.11	>20	4.19 ± 0.80
**7d**	−	4-Cl-C_6_H_4_	2.56 ± 0.61	2.67 ± 0.89	2.29 ± 0.86
**7f**	−	2-furyl	6.37 ± 3.40	6.65 ± 2.20	5.22 ± 0.75
**7g**	−	2-thienyl	3.74 ± 1.95	6.19 ± 1.49	3.26 ± 0.39
**9c**	−	4-CH_3_O-C_6_H_4_	4.24 ± 2.38	5.06 ± 2.23	3.92 ± 2.15
**9d**	−	4-Cl-C_6_H_4_	2.48 ± 2.21	3.55 ± 2.44	2.93 ± 2.10
**9e**	−	4-F-C_6_H_4_	3.46 ± 1.44	4.92 ± 2.51	3.83 ± 0.52
**9g**	−	4-*t*-Bu-C_6_H_4_	5.43 ± 0.90	7.39 ± 1.71	4.33 ± 1.11
**9h**	−	2-naphthyl	6.59 ± 0.81	>20	6.44 ± 1.96

* Values are the average of three independent determinations with standard deviations.
